# GDF15 propeptide promotes bone metastasis of castration-resistant prostate cancer by augmenting the bone microenvironment

**DOI:** 10.1186/s40364-024-00695-6

**Published:** 2024-11-25

**Authors:** Gaku Yamamichi, Taigo Kato, Noriaki Arakawa, Yoko Ino, Takeshi Ujike, Kosuke Nakano, Yoko Koh, Yuichi Motoyama, Hidetatsu Outani, Shohei Myoba, Yu Ishizuya, Yoshiyuki Yamamoto, Koji Hatano, Atsunari Kawashima, Shinichiro Fukuhara, Hiroji Uemura, Seiji Okada, Eiichi Morii, Norio Nonomura, Motohide Uemura

**Affiliations:** 1 Department of Urology, Osaka University Graduate School of Medicine, 2-2 Yamadaoka, Suita, Osaka 565-0871 Japan; 2 Division of Medicinal Safety Science, National Institute of Health Sciences, 3-25-26, Tonomachi, Kawasaki, Kanagawa 210-9501 Japan; 3 Advanced Medical Research Center, Yokohama City University, 3-9 Fukuura, Yokohama, Kanagawa 236-0004 Japan; 4 Department of Pathology, Osaka University Graduate School of Medicine, 2-2 Yamadaoka, Suita, Osaka 565-0871 Japan; 5 Department of Orthopedic Surgery, Osaka University Graduate School of Medicine, 2-2 Yamadaoka, Suita, Osaka 565-0871 Japan; 6 Bioscience Division, Research and Development Department, Tosoh Corporation, 2743-1 Hayakawa, Ayase, Kanagawa 252-1123 Japan; 7 Departments of Urology and Renal Transplantation, Yokohama City University Medical Center, 4-57 Urafunechou, Yokohama, Kanagawa 232-0024 Japan; 8 Department of Urology, Iwase General Hospital, 20 Kitamachi, Sukagawa, Fukushima, 962-8503 Japan; 9 Department of Urology, Fukushima Medical University School of Medicine, 1 Hikarigaoka, Fukushima, Fukushima, 960-1295 Japan

**Keywords:** Castration-resistant prostate cancer, Bone metastasis, GDF15, Biomarker

## Abstract

**Background:**

Bone metastasis (BM) is a common and fatal condition in patients with castration-resistant prostate cancer (CRPC). However, there are no useful blood biomarkers for CRPC with BM, and the mechanism underlying BM is unclear. In this study, we investigated precise blood biomarkers for evaluating BM that can improve the prognosis of patients with CRPC.

**Methods:**

We comprehensively examined culture supernatants from four prostate cancer (PCa) cell lines using Orbitrap mass spectrometry to identify specific proteins secreted abundantly by PCa cells. The effects of this protein to PCa cells, osteoblasts, osteoclasts were examined, and BM mouse model. In addition, we measured the plasma concentration of this protein in CRPC patients for whom bone scan index (BSI) by bone scintigraphy was performed.

**Results:**

A total of 2,787 proteins were identified by secretome analysis. We focused on GDF15 propeptide (GDPP), which is secreted by osteoblasts, osteoclasts, and PCa cells. GDPP promoted the proliferation, invasion, and migration of PC3 and DU145 CRPC cells, and GDPP aggravated BM in a mouse model. Importantly, GDPP accelerated bone formation and absorption in the bone microenvironment by enhancing the proliferation of osteoblasts and osteoclasts by upregulating individual transcription factors such as *RUNX2*, *OSX*, *ATF4*, *NFATc1*, and *DC-STAMP*. In clinical settings, including a total of 416 patients, GDPP was more diagnostic of BM than prostate-specific antigen (PSA) (AUC = 0.92 and 0.78) and the seven other blood biomarkers (alkaline phosphatase, lactate dehydrogenase, bone alkaline phosphatase, tartrate-resistant acid phosphatase 5b, osteocalcin, procollagen I N-terminal propeptide and mature GDF15) in patients with CRPC. The changes in BSI over time with systemic treatment were correlated with that of GDPP (*r* = 0.63) but not with that of PSA (*r* = -0.16).

**Conclusions:**

GDPP augments the tumor microenvironment of BM and is a novel blood biomarker of BM in CRPC, which could lead to early treatment interventions in patients with CRPC.

**Supplementary Information:**

The online version contains supplementary material available at 10.1186/s40364-024-00695-6.

## Introduction

Prostate cancer (PCa) is currently the most commonly diagnosed malignancy in the male population in more than half of the countries worldwide, with an incidence of approximately 1.4 million cases per year, and is the second leading cause of cancer-related deaths among men [1, 2]. Although first-line treatments, including androgen deprivation therapy for metastatic hormone-sensitive prostate cancer (mHSPC), are initially highly effective in decreasing the levels of the standard indicator of PCa progression, namely, prostate-specific antigen (PSA), and in shrinking tumors, therapeutic resistance is almost universal, and the disease often progresses to metastatic castration-resistant prostate cancer (mCRPC).

Generally, PCa has the highest incidence of bone metastases (BM) among cancers, with 6–8% of new PCa patients having BM at first diagnosis [3, 4] and more than 90% of patients with CRPC developing BM [5]. However, to date, no useful blood biomarkers for diagnosing and monitoring the BM due to CRPC have been identified [6–8] because CRPC mainly consists of androgen-independent PCa [9, 10]. In addition, approximately 20% of PCa cases are accompanied by neuroendocrine alterations during the treatment course [11], which is associated with difficulty in assessing disease progression solely based on PSA levels [12–15], suggesting that the evaluation of PSA levels is not sufficient to accurately predict BM status [16, 17]. Bone scintigraphy is often used to evaluate BM volume in patients with CRPC in combination with laboratory parameters, including alkaline phosphatase (ALP) [18]. However, bone scintigraphy has several disadvantages, such as high cost and radiation exposure, resulting in difficulty in frequent measurements [7]. In this context, accurate and noninvasive biomarkers of BM are urgently required.

Growth differentiation factor 15 (GDF15), also known as macrophage inhibitory cytokine 1 and NSAID-activated gene-1, is a member of the transforming growth factor β (TGF-β) superfamily [19]. Among various types of cancers, PCa exhibits the highest *GDF15* transcript expression [20]. The *GDF15* gene encodes a 308-aa peptide (pre-pro-GDF15) consisting of an N-terminal signal peptide, a mature domain (mGDF15), and a propeptide domain, which we named the GDF15-derived propeptide GDPP. The pro-GDF15 precursor is secreted as a homodimer from the endoplasmic reticulum. The active mature form, mGDF15, is released via the proteolytic cleavage of dimeric pro-GDF15 at a furin-like site (RXXR) [21–23]. A recent study showed that mGDF15 binds to glial cell-derived neurotrophic factor family receptor alpha-like, is involved in PI3K/Akt/mTOR pathway activation, and participates in various physiological processes such as weight loss. In contrast, the GDPP domain is thought to be involved in the recognition and disposal of pre-pro-GDF15, depending on whether it is correctly folded, and processing of the precursor within the cell [24]. However, no attention has been paid to the free GDPP domain released after its detachment from the mGDF15 domain, resulting in a lack of reports on the physiological functions and extracellular dynamics of free GDPP.

In this study, we aimed to identify a convenient and accurate diagnostic biomarker that can enable the monitoring of BM in patients with CRPC and found that the newly identified protein “GDPP” promotes PCa progression and bone formation and resorption via the upregulation of transcription factor expression in the bone microenvironment, suggesting that plasma GDPP is a novel biomarker that reflects BM status more accurately than PSA in patients with CRPC and BM. Collectively, we believe that compared with traditional imaging tests, GDPP detection will reshape the diagnosis of BM.

## Materials and methods

### Secretome analysis

Proteomic analysis of culture medium from the PCa cell lines, described in Supplemental Materials “Cell culture and maintenance’’, was performed as previously described [25]. In brief, the PCa cell lines were cultured under the recommended conditions until they reached 60% confluency. Then, the media were replaced with serum-free media, and cells were incubated for 48 h. The culture media were then collected and lyophilized. The lyophilized media were dissolved in 10 mM ammonium bicarbonate containing 4 M urea, and proteins were desalted by acetone precipitation. The precipitated protein was resuspended in 25 mM ammonium bicarbonate containing 4 M urea and 0.1% RapiGest detergent (Nihon Waters, Tokyo, Japan) and subsequently digested with trypsin for 16 h at 37°C after reduction, alkylation and dilution. The resulting peptides were desalted using C18 Stage Tips [26] and analyzed on an LTQ Orbitrap Velos (Thermo Fisher Scientific) equipped with a reverse-phase LC system. Peptides were detected sequentially in positive ion mode for MS/MS in data-dependent scanning mode and identified using Proteome Discoverer 2.5 software (Thermo Scientific) and the Swiss-Prot human database (www.uniprot.org/proteomes/UP000005640) with the following parameters: enzyme, trypsin; peptide mass tolerance, ± 5 ppm; fragment mass tolerance, ± 0.5 Da; maximum missed cleavage sites, 2; variable modifications: oxidation of methionine, acetylation and/or loss of methionine at N-terminus; and static modification: carbamidomethylation of cysteine. Mass spectrometry proteomics data were deposited to the ProteomeXchange Consortium (PXD045369, http://www.proteomexchange.org/) via the jPOST partner repository (JPST002261, https://jpostdb.org/).

In this study, we counted a protein as “present protein” when it is identified in at least one out of the three measurements of the culture media under the condition of false discovery rate < 1%, as described previously [25].

### Structure modeling with AlphaFold2

Structure predictions for pre-pro-GDF15, GDPP and mGDF15 were generated by the AlphaFold2 (https://colab.research.google.com/github/sokrypton/ColabFold/blob/main/AlphaFold2.ipynb, accessed on 4 June 2023) model using the relevant online resources with their default settings [27, 28].

### Open source RNA-sequencing analysis

RNA-seq transcriptome data of various cancer patients, including 493 PCa, 407 bladder cancer, 510 renal cell carcinoma, 1082 breast cancer, 484 lung cancer, 592 colon cancer, 443 melanoma, 412 gastric cancer, 366 liver cancer, 527 uterine cancer, 181 esophageal cancer, 515 head and neck carcinoma, 514 glioma and 177 pancreatic cancer patients, were downloaded from the TCGA database in 2018.

### Human sample collection and data

Between December 2012 to December 2022, a total of 416 patients were included in this study. All patients were confirmed to have adenocarcinoma by prostate needle biopsy at the time of diagnosis. The blood samples were collected once from 30 healthy donors, 60 localized PCa patients, 30 mHSPC patients with BM, 15 mCRPC patients without BM, and 80 mCRPC patients with BM. We also collected blood samples from 22 mCRPC patients with BM, before and after systemic therapy, and from 179 localized PCa patients, before and after radical prostatectomy for the analysis of GDPP dynamics.

In addition, we also obtained primary tumors, tissues of BM, and tissues of lymph nodes metastasis from the four mCRPC patients.

Whole blood (2.0–7.0 ml) was collected directly into Venoject II EDTA-2Na tubes (TERUMO) for plasma samples, and whole blood (2.0–7.0 ml) was collected directly into Venoject II tubes (TERUMO) for serum samples. Within three hours of collection, all plasma samples were centrifuged sequentially at 900 and 20,000 × g for 10 min each, and the supernatants were stored at − 80 °C as plasma, as described previously [29, 30]. All serum samples were centrifuged at 3000 rotations per minute (rpm) for 5 min, and the supernatants were stored at -80 °C as serum. Serum PSA (Beckman Coulter), ALP (Shino-Test Corporation), BAP (IDS, Inc.), TRACP 5b (Nittobo Medical), LDH (FUJIFILM Wako Pure Chemical Corporation), OC (Tosoh), mGDF15 (R&D Systems) and PINP (USCN) levels were measured in the same blood samples. Bone scan index (BSI) was assessed within two months of both blood collection time points. Before the collection of human blood from patients, written informed consent was obtained from each patient, and all experiments were carried out following institutional ethical regulations and guidelines under protocols approved by the Institutional Review Board of Osaka University Hospital (# 13397-19).

### Bone scintigraphy

All PCa patients were injected intravenously with 740 MBq of 99mTc MDP to evaluate the existence of BM. Three hours after injection, a whole-body bone scan was performed with a gamma camera equipped with a low-energy high-resolution parallel hole collimator in anterior and posterior views. The raw image data set was analyzed with the software package BONENAVI version 2, based on a personal database in Japan. This CAD system was used to calculate the BSI, which was calculated as a percentage of the sum of all spots classified as bone metastases in the patient’s body. When the attending physician deemed it necessary especially for CRPC patients, it was taken about once every three month and their data was retrospectively analyzed.

### Immunofluorescence staining

LNCaP and PC3 cells were seeded in 2-well chamber slides (5712-002, IWAKI) at a density of 3 × 10^5^ cells/1.5 ml/well and 1 × 10^5^ cells/1.5 ml/well, respectively and incubated overnight at 37 °C in a humidified atmosphere containing 5% CO_2_. The cells were then washed with PBS and fixed with 4% paraformaldehyde for 15 min on ice. After permeabilization with 0.1% Triton X-100 (87361, Muto Pure Chemicals Co., Ltd.) in PBS at room temperature for 15 min, the cells were incubated with primary antibodies diluted in PBS-T overnight at 4 °C. The primary antibodies used were a rabbit anti-GDPP polyclonal antibody (HPA011191, Sigma‒Aldrich, 1:200) and a mouse anti-mGDF15 monoclonal antibody (sc-515675, Santa Cruz Biotechnology, 1:50). After PBS washes, the slides were incubated with the appropriate secondary antibodies, Alexa Fluor 488 goat anti-mouse secondary antibody (A-11001, Invitrogen) and Alexa Fluor 568 goat anti-rabbit secondary antibody (A-11011, Invitrogen), both diluted in PBS-T (1:500), for 1.5 h at room temperature. The slides were then washed with PBS-T at 22 °C and counterstained with ProLong Gold Antifade reagent with DAPI (P36931, Invitrogen). The stained LNCaP cells were examined using a fluorescence microscope (BZ-X710, KEYENCE). Rabbit polyclonal IgG (NBP2-24891, Novus) and mouse monoclonal IgG (ab18469, Abcam) were used as isotype controls for the respective antibodies.

### Establishment of an ELISA system to measure GDPP

Anti-GDPP monoclonal antibodies targeting the GDF15 propeptide, namely, GD11-13 and GD01-62, were generated using a plasmid DNA immunization method, as we reported previously [31]. These antibodies specifically recognize the central region of GDPP. To detect GDPP, a combination of GD11-13, immobilized on magnetic microparticles, and GD01-62, labeled with alkaline phosphatase, was employed. AIA-CL reagent (Tosoh) was developed based on the two-step sandwich enzyme immunoassay technique. Using the fully automated chemiluminescent enzyme immunoassay system (AIA-CL2400, Tosoh), sample dispensing, immunoreaction, B/F separation, substrate addition, and luminescence detection were performed automatically, and results were obtained in approximately 15 min.

### Cell culture and maintenance

LNCaP and DU145 cells were purchased from RIKEN BRC CELL BANK, 22Rv1 and PC3 cells were purchased from the American Type Culture Collection (ATCC). All cell lines were maintained in basal culture medium (RPMI1640) (Nacalai Tesque) with 10% fetal bovine serum (FBS), 100 U/mL penicillin G, and 0.1 µg/mL streptomycin in a humidified incubator set to 37 °C and 5% CO_2_. PC3-Luc2 cells were also purchased from the ATCC and maintained in basal culture medium [Ham’s F-12 K Kaighn’s medium, Gibco™; 10% FBS; 8 µg/mL Blasticidin S (Invitrogen)] in a humidified incubator set to 37 °C and 5% CO2. MC3T3-E1 cells (RIKEN BRC Cell Bank) were maintained in basal culture medium (αMEM, Nacalai Tesque) with 10% FBS, 100 U/mL penicillin G, and 0.1 µg/mL streptomycin, MLO-Y4 cells (Kerafast) were cultured on type I collagen-coated dishes (Corning) and maintained in basal culture medium (αMEM with 5% heat inactivated FBS, 5% calf serum, 100 U/mL penicillin G, and 0.1 µg/mL streptomycin), and OSC14C cells (Cosmo Bio) were suspended in osteoclast culture medium (OSCMW and OSCMM, Cosmo Bio). HOB (PromoCell, lot number #469Z022, from cancellous bone/femoral head tissue collected from a 78-year-old Caucasian man) was cultured in osteoblast growth medium (C-27001, PromoCell), and OSC15C (Cosmo Bio, lot number #VJ2-F-OSH) was cultured in osteoclast wash medium (OSCMW, Cosmo Bio) and growth medium including receptor activator of NF-κB ligand and macrophage-colony stimulating factor (OSCMW, Cosmo Bio). HOB was used for functional analysis with a maximum of five passages allowed for cell culture. OSC14C and OSC15C differentiation into mature osteoclasts was confirmed by TRAP staining. A cell scraper (99002, Techno Plastic Products) was used to scrape off the cells.

### Analysis of secreted proteins in cell culture media

To analyze the secreted proteins in culture media, we seeded 5 × 10^5^ cells in 2 ml of serum-free medium into a 6-well dish and collected the culture medium 24 h after seeding. This medium was passed through a filter (Millex-GV, SLGVR33RS, Merck), and the filtrate was collected after centrifugation at 6000×g for 30 min using a centrifugal concentrator (Vivaspin, VS2091, SARTORIUS). The GDPP concentration in the culture medium for each sample was measured in triplicate. In total, 5 × 10^5^ LNCaP cells were seeded in 6-well dishes, and the medium was changed 24 h later. Then 25 µM furin inhibitor (#14965, Cayman Chemical) was added for 24 h, and the whole-cell lysate and culture medium were collected.

### Sodium dodecyl sulfate‒polyacrylamide gel electrophoresis (SDS‒PAGE) and Western blotting

For SDS**‒**PAGE, sample buffer containing 10% 2-mercaptoethanol was added to whole-cell lysates, generated using RIPA Lysis Buffer (Santa Cruz Biotechnology), or culture media, and proteins were resolved on 10% polyacrylamide mini gels (TEFCO). Afterward, proteins were transferred onto a polyvinylidene difluoride membrane using a semidry transfer system (Thermo Fisher Scientific). The membrane was then probed with the indicated specific antibodies that were utilized for immunological analysis: GDPP (1:1000, HPA011191, Sigma‒Aldrich), mGDF15 (1:1000, LS-C383688, LSBio), and β-actin (1:5000, 4967 S, Cell Signaling Technology). The membrane was incubated with a horseradish peroxidase-conjugated secondary antibody against rabbit immunoglobulin (1:5000, Cell Signaling Technology). Finally, the membrane was subjected to detection with enhanced chemiluminescence western blotting detection reagents (Nacalai Tesque) and visualized using the ChemiDoc XRS Plus system (Bio-Rad) as a chemiluminescence detector.

### Development of human recombinant GDPP

The sequence of human GDPP with a Strep-tag at the N-terminus was cloned and inserted into an expression vector, and the resulting plasmid was amplified and utilized to transfect Expi293 mammalian cells for Strep-GDPP expression. The transfected cells were cultured, and the culture medium was collected. The recombinant GDPP protein was purified from the culture medium using a Strep-tag purification kit (IBA Lifesciences) according to the manufacturer’s instructions.

### Immunohistochemical studies

Both human and mouse bone metastasis specimens were demineralized using Tris-ethylenediaminetetraacetic acid (EDTA) demineralization solution until tissue softening was observed, followed by paraffin fixation. Immunohistochemical staining was performed in 4 μm-thick paraffin-embedded tissue samples. The human sample sections and sections of xenograft bone tumor samples were treated with EDTA buffer (pH 9.0) and activated by warming at 125 °C for 30 s using a Pascal pressure chamber (S2800, Dako) for antigen activation treatment. Endogenous peroxidase activity was blocked by incubating the sections with 0.3% hydrogen peroxide for 5 min, followed by overnight incubation with primary antibodies against GDPP (1:200; HPA011191, Sigma‒Aldrich), furin (1:50; bsm-54283R, Bioss antibodie), CD8 (1:100; ab17147, abcam), CD80 (1:1000; ab134120, abcam) and Ki67 (1:200; ab16667, abcam) at 4 °C, and staining was performed using DAB substrate (MK210, TaKaRa). Finally, the sections were counterstained with hematoxylin. For IHC analysis of pro-GDF15 and furin, we caluated the IHC score (= intensity score × percentage score cells in a 400× field of view) using three different random fields per sample. Intensity score was evaluated according to the staining intensity (0: negative, 1: weak, 2: moderate, and 3: strong); percentage score was evaluated based on the percentage of stained cells (0: 0%, 1: 1–25%, 2: 26–50%, 3: 51–75%, and 4: 76–100%) [32]. For IHC analysis of Ki67, the ratio of positive cells to tumor cells was counted in a 400× field of view and the averages of the ratio was calculated using three different random fields per sample, as we reported previously [33]. Following this, the averages of the score and ratio was calculated using three different random fields per sample. In the mouse tibial bone tissue specimens, antigen activation treatment was performed with 3-fold diluted Proteinase K Ready-to-use (S3020, Dako). The sections were incubated overnight at 4 °C with a primary antibody against the osteoblast marker OC (M188, Takara, diluted 100 times), followed by incubation with secondary anti-rat antibody (714311, Nichirei Bioscience, Inc.). Staining was performed using DAB substrate (MK210, TaKaRa). Osteoclasts were stained using a commercially available TRAP Staining Kit (AK04F, Cosmo Bio). Osteoclasts were identified as TRAP-positive multinucleated (three or more nuclei) cells, and osteoblasts and osteoclasts were counted on the trabecular bone matrix surface in three randomly selected fields of view using light microscopy (BZ-X710, KEYENCE).

### RNA interference

For knockdown of *GDF15* using small interfering RNA (siRNA), cells were transfected with 10 nM of either targeting FlexiTube GeneSolution (GS9518, Qiagen) or negative control Stealth RNAi™ (12935112, Invitrogen) using Lipofectamine^®^ RNAiMAX Reagent (13778075, Invitrogen) for 24 h. Then, the medium containing siRNA and transfection reagent was replaced with fresh medium. Following validation of *GDF15* knockdown confirmed by western blotting method, functional assays were performed.

### Cell proliferation assay

PC3, LNCaP and HOB transfected with either siRNA targeting *GDF15* or negative control for 72 h were reseeded in medium supplemented with 10% FBS in 96-well plates at 1 × 10^3^ cells/100 µL/well, 1 × 10^3^ cells/100 µL/well and 1.3 × 10^3^ cells/100 µL/well, respectively; DU145 cells were seeded in the same medium in 96-well plates at 1 × 10^3^ cells/100 µL/well. The cells were incubated for 1 h at 37 °C in a humidified 5% CO_2_ atmosphere, and then 0.1 µl MT Cell Viability Substrate (G9712, Promega) and 0.1 µl NanoLuc^®^ Enzyme (G9712, Promega) were added to each well. Luminescence was measured with a GloMax^®^ Explorer System (GM3510, Promega) according to the manufacturer’s instructions after 24, 48, and 72 h in a humidified incubator set to 37 °C and 5% CO_2_; this timepoint was set as 0 h, and GDPP was added at this point. The assay was repeated three times for each experimental group.

### Wound-healing assay

PC3 transfected with either siRNA targeting *GDF15* or negative control siRNA for 72 h were reseeded in 6-well plates at 6 × 10^5^ cells/2 mL/well, and DU145 cells were seeded in 6-well plates at 6 × 10^5^ cells/2 mL/well. Cells were grown to a monolayer, and a wound was created by scraping the cell layer using a sterile 200-µL yellow pipette tip when the cells reached approximately 90% confluence. Detached cells were removed by washing plates with PBS and adding fresh culture medium supplemented with 10% FBS to each plate. Cells were treated with or without GDPP at this point and then incubated at 37 °C with 5% CO_2_. Cell migration was evaluated using a fluorescence microscope (BZ-X710, KEYENCE) at 0 h and 20 h after wound generation and quantified by measuring the size of the recovered area using ImageJ 1.53e. The assay for each experimental group was repeated three times.

### Cell invasion assay

PC3 and LNCaP transfected with either siRNA targeting *GDF15* or negative control siRNA for 72 h and DU145 cells were seeded into the upper chambers of Corning^®^ BioCoat™ Matrigel Invasion Chambers (pore size—8 μm) (354480, Corning) (5 × 10^4^ cells/well) in serum-free medium, with medium supplemented with 10% FBS present in the lower chamber. GDPP was added immediately after cell seeding. After 36 h of incubation and in LNCaP, after 72 h of incubation at 37 °C and 5% CO_2_, the cells that had penetrated into the Matrigel matrix were fixed and stained using a Diff-Quik stain kit (Sysmex, Kobe, Japan), and cell invasion was quantified using a fluorescence microscope (BZ-X710, KEYENCE). The analysis was repeated three times for each experimental group.

### Quantitative real-time PCR

Total RNA was extracted from HOB (treated with or without GDPP for 3 days) and OSC15C cells (treated with or without GDPP for 12 days) using the ISOSPIN Cell & Tissue RNA Kit (NIPPON GENE). After verification of RNA quality by NanoDrop One (Thermo Fisher Scientific), the RNA was subjected to cDNA synthesis using the Prime Script RT reagent Kit (Perfect Real Time; TaKaRa Bio). Quantitative RT‒PCR was performed using the Thermal Cycler Dice Real Time System (TP800; Takara) and THUNDERBIRD™ Next SYBR^®^ qPCR Mix (TOYOBO). Target gene expression was normalized to that of the housekeeping gene *GAPDH* using the delta-delta Ct method. The primers used for the PCRs are listed in Table S1, and all PCRs were performed in triplicate for each sample.

### ALP staining and assessment of activity

HOB was seeded in 24-well plates and cultured on type I collagen-coated dishes (Corning) at 1 × 10^5^ cells/0.5 ml/well with or without GDPP. After 5 days of incubation, for ALP staining, the cells were washed with PBS and fixed for 20 min with 10% formalin at room temperature. After fixation, the cells were incubated with an Alkaline Phosphatase Staining kit (AK20, Cosmo Bio Co., Ltd.) for 20 minutes at 37°C according to the manufacturer’s instructions. The total percentage of ALP^+^ cells was determined using a fluorescence microscope. To measure ALP activity, HOB was seeded to 24-well plates and incubated with or without GDPP for 5 days. Then, WCLs were obtained as stated in the Supplementary Materials section “Sodium dodecyl sulfate‒polyacrylamide gel electrophoresis (SDS‒PAGE) and western blotting’’, and ALP activity was measured using p-nitrophenylphosphate as the substrate for the alkaline phosphatase test (QFAP-100, BioAssay System) according to the manufacturer’s instructions. The optical density (OD) of WCLs was measured in a 96-well plate at 405 nm with a microplate reader (iMARK™, Bio-Rad). The assay was repeated three times for each experimental group.

### Alizarin Red S staining and bone mineralization quantification

Human primary osteoblasts (HOB) was seeded in 24-well plates and cultured on type I collagen-coated dishes (354408, Corning) at 3 × 10^4^ cells/0.5 ml/well using Osteoblast Mineralization Medium (C-27020, PromoCell) with or without GDPP for 21 days. The cells were subsequently washed with PBS, fixed, stained and digested with an Alizarin Red S staining kit (BMK-R009, BMK, Bio Mirai Kobo). The total area of staining was quantified using a fluorescence microscope, and after staining, the OD of the eluted Alizarin Red S solution was measured in a 96-well plate at 405 nm with a microplate reader (iMARK™, Bio-Rad). The assay was repeated three times for each experimental group.

### TRAP staining and resorption pit assay

Human-derived primary osteoclast precursor cells (OSC15C) was seeded in 24-well plates at 2 × 10^5^ cells/0.5 ml/well with or without GDPP for 12 days. TRAP staining was performed using a TRAP staining kit (AK04F, Cosmo Bio) according to the manufacturer’s instructions, and the total number of TRAP-positive cells with ≥ 3 nuclei was determined using a fluorescence microscope as the average of five randomly observed fields of view. The resorption pit assay was performed using the Bone Resorption Assay Kit (BRA-48KIT, PG Research). OSC15C cells were seeded in a 48-well Bone Resorption Assay Plate 48 (BRA-48P, PG Research) at 8 × 10^4^ cells/0.5 ml/well with or without GDPP for 12 days. The bone resorption area was quantified using a fluorescence microscope as the average of five randomly observed fields of view. The assay was repeated three times for each experimental group.

### Animal experiments with bone metastasis of prostate cancer

Male NOD.CB17-Prkdc^SCID^/J mice, 5–6 weeks of age, were purchased from Japan Charles River Laboratories, Inc. All mice were euthanized under anesthesia using isoflurane. For intratibial implantation, 1 × 10^6^ PC3-Luc2 cells, purchased from ATCC, were suspended in 5 µl of VitroGel Hydrogel Matrix (VHM01S, TheWell Bioscience LLC) and 5 µl of phosphate-buffered saline (PBS) [34, 35]. Mice were anesthetized with isoflurane, and the cell suspension was directly injected into the intramedullary cavity of the right tibia. The cavity was reached by drilling into the cortical bone of the tibial tuberosity using a 22 G needle (NN-2232R, TERUMO) with a 1 ml 29G syringe containing a needle (08299, NIPRO). Then, the skin was closed with a 6–0 suture. Mice in the treatment group were subcutaneously injected with recombinant GDPP (refer to the section “Development of human recombinant GDPP’’) dissolved in saline at a concentration of 0.1 mg/kg every other day from day 1 to day 50. Control mice were injected with an equal amount of saline from day 1 to day 50, and subsequent tumor growth was evaluated weekly with bioluminescence analysis via an In Vivo Imaging System (IVIS^®^ Lumina II, Caliper). The mice were randomized into two groups for experiments: the control (n = 10) and GDPP groups (n = 10).

### Micro-CT and IVIS imaging

The mice were imaged to visualize luciferase activity immediately after injection and were monitored weekly using IVIS^®^ imaging. Bioluminescence images of tumor-bearing mice were acquired with an IVIS Spectrum 10 min after intraperitoneal injection of D-luciferin (XLF-1, Summit Pharmaceutical International Corporation, 100 mg/kg), with an exposure time of 10 s. As a quality control measure, the photon flux was measured by quantifying the number of highlighted pixels within a circular region of interest (ROI) for each mouse in the supine position. These values were then normalized to the signal intensity obtained immediately after xenografting in the same area (day 0) of each mouse. Thus, all mice had an arbitrary starting normalized bioluminescence signal intensity of 1. This normalization was performed using Living Image^®^ software version 4.2 (Caliper Life Sciences, Inc.) following the manufacturer’s instructions. Bioluminescence imaging was employed to assess tumor burden and the localization of PC3-Luc2 cells. The bone-destructive phenotype caused by PC3-Luc2 cells was visually evaluated macroscopically. Additionally, three-dimensional images were constructed to confirm the presence of bone infiltration using R_mCT2 software (Rigaku) for micro-CT imaging. The volume of bone tumors 50 days after the administration of PC3-LuC2 was measured using µCT and calculated as long diameter × long diameter × short diameter × 1/2 [36].

### Statistics

The statistical analyses were performed using JMP Pro (v.17.0.0; SAS Institute, NC, USA). Univariate analysis included two-tailed Student’s t-test and the Mann‒Whitney U test. Multiple comparisons were assessed using the Tukey‒Kramer method to compare several treatments. Univariate and multivariate analyses were performed using the Cox proportional hazards regression model or logistic regression analysis. Spearman’s rank correlation coefficient was used as a measure of the strength of the correlation between the two variables. Statistical significance was defined as *p* < 0.05. The optimal cutoff value for diagnosis was determined from the receiver operating characteristic curve using the Youden index, and recall rate, F1 score, precision, sensitivity and specificity for diagnosis were calculated based on each optimal cutoff value. The cutoff values for the parameters used in the diagnostic and prognostic analysis are the respective medians.

## Results

### GDF15 propeptide is a unique secreted peptide in PCa cells

To identify candidate proteins secreted by PCa cells that may be useful as blood biomarkers, we first performed secretome analysis of the culture media from four PCa cell lines (LNCaP, 22Rv1, PC3, and DU145) and proteomic analysis of the peptides obtained. We identified 17,798 peptides from 2,787 proteins in all culture media from PCa cell lines. Among these secreted proteins, we focused on GDF15, which was identified in three of the four cell lines (LNCaP, 22Rv1, and PC3) (Fig. S1A) because in neuroendocrine prostate cancer (NEPC), GDF15 is highly expressed and PSA levels do not clearly reflect disease progression [37, 38].

Additionally, our secretome analysis revealed peptides annotated to the mGDF15 domain in the C-terminal region and multiple peptides located in the GDPP domain of the N-terminal propeptide in the culture media (Fig. S1B). GDF15 secreted more unique peptides than PSA from the three different PCa cell lines (LNCaP, 22Rv1 and PC3) (Table S2), and considering that there are no reports regarding the function of GDF15 propeptide in human blood, we focused on GDPP. Three-dimensional (3D) structural predictions showed spatial connectivity between the signal peptides, mGDF15 and GDPP (Fig. S1C) [27, 28]. In addition, analysis of bulk RNA-seq data from The Cancer Genome Atlas (TCGA) database showed that the expression levels of *GDF15* were the highest, and those of *Furin*, which cleaves the junction between the GDPP domain and the remainder of the mGDF15 sequence, were relatively higher in PCa than in other cancer types (Fig. S1D, S1E).

### GDPP is more useful than other blood biomarkers in CRPC patients with bone metastasis

**Fig. 1:**
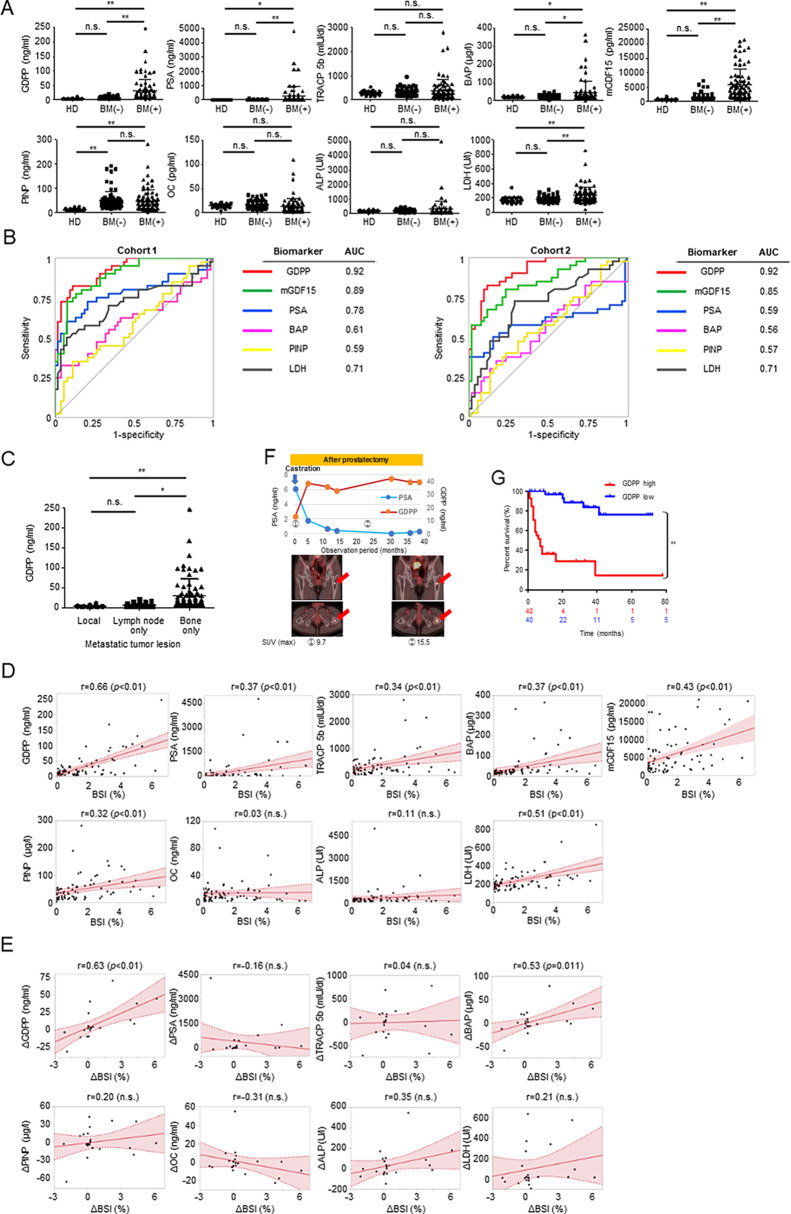
Real-world data on GDPP in CRPC patients with BM. (**A**) A comparison of GDPP, PSA, TRACP 5b, BAP, mGDF15, PINP, OC, ALP, and LDH levels in healthy donors (*n* = 30) versus CRPC patients with and without BM (*n* = 80 and 75, respectively). The data show significantly elevated levels of GDPP, PSA, BAP, mGDF15 and LDH in patients with BM. GDPP: Growth differentiation factor 15 propeptide, Data are expressed as the mean ± standard deviation (SD), and statistical analyses were performed using the Tukey‒Kramer method (* *p* < 0.05, ** *p* < 0.01; n.s., not significant). (**B**) GDPP had the best AUC when comparing the diagnostic performance of each blood biomarker for BM in CRPC patients in each of the two randomized cohorts. (**C**) Comparison of blood GDPP levels by site of metastasis in PC (Local only: *n* = 60, Lymph node metastatic CRPC only: *n* = 12, Bone metastatic CRPC only: *n* = 31). (**D**) The analysis of the relationship between the BSI and GDPP, PSA, TRACP 5b, BAP, mGDF15, PINP, OC, ALP, or LDH in CRPC patients with BM is shown, together with the comparison of the strength of the correlation between each biomarker and the BSI (*n* = 80). Statistical analyses were performed using Spearman’s rank correlation coefficient. (**E**) The relationships between the change in BSI (ΔBSI) and the changes in many blood biomarkers during systemic treatment in CRPC patients with BM showed that the change in GDPP (ΔGDPP) correlated best with the change in the BSI (*n* = 22). Statistical analyses were performed using Spearman’s rank correlation coefficient. (**F**) This panel shows data from a representative patient who underwent longitudinal monitoring. During the clinical course of the patients, the plasma GDPP levels, rather than PSA levels, reflected the volume of BM revealed by PSMA PET. Red arrow indicates the solitary BM location from PCa. (**G**) Kaplan‒Meier analysis of the OS of CRPC patients with BM stratified by GDPP value; statistical analyses were performed using the log-rank test (** *p* < 0.01)

To examine the clinical relevance of GDPP, we measured GDPP levels in patients with CRPC for whom PSA levels were not considered reliable. The clinical characteristics of the patients are summarized in Table 1 (*n* = 185). In this study, the pathological results of prostate cancer patients were all adenocarcinomas. The data showed that there were significant increases in the plasma levels of GDPP and serum levels of PSA, mGDF15, and bone turnover markers such as BAP and LDH in CRPC patients with BM compared with PCa patients without BM (Fig. 1A). Notably, receiver operating characteristic (ROC) analysis revealed that GDPP had the strongest diagnostic ability for BM of CRPC among these blood markers in the two cohorts, with an area under the curve (AUC) of 0.92 (Fig. 1B, Tables S3, S4). Furthermore, we found a significant increase of GDPP levels in CRPC patients only with BM (*n* = 31) compared to those only with lymph node metastasis (*n* = 12) and local prostate cancer (*n* = 60) (*p* = 0.0124 and *p* < 0.01, respectively, Fig. 1C), suggesting GDPP levels particularly increase in patients with BM.

**Table 1:** Patient baseline characteristics and blood biomarker levels. All continuous data use median and range

	Healthy donors *n* = 30	Localized PCa *n* = 60	mCRPC (BM-) *n* = 15	mCRPC (BM+) *n* = 80
Age (years)	63 (37–74)	69 (52–79)	73 (59–86)	74 (51–88)
Gleason Score				
< 8 ≥ 8	-	53 7	6 9	16 64
pT stage pT2 pT3	-	48 12	-	-
Metastasis site				
Bone Lymph node Lung Liver Bladder Ureter	-	-	0 12 0 2 0 1	80 44 8 2 1 0
PSA (ng/ml)	0.96 (0-8.5)	6.5 (2.6–45.5)	5.6 (0-110.8)	17.7 (0-4782)
ALP (U/l)	183 (45–292)	196 (107–442)	186 (60–334)	187 (37-4987)
LDH (U/l)	172 (118–340)	175 (120–312)	183 (151–280)	211 (115–853)
OC (ng/ml)	15.2 (4.5–22.0)	14.7 (3.6–35.8)	14.4 (6.1-38.31)	7.8 (1.2-109.5)
BAP (µg/l)	18.4 (13.9–30.7)	22.2 (10.5–49.4)	19.4 (3.7–45.3)	22.5 (0.8-364.9)
PINP (ng/ml)	11.9 (6.5–24.6)	36.4 (14.8–72.2)	95.6 (21.5-192.2)	37.0 (3.9-280.9)
TRACP 5b (mIU/dl)	286.2 (141.8-532.9)	276.0 (117.1-582.6)	357.0 (159.9-953.4)	237.0 (17.9–2795)
mGDF15 (pg/ml)	626.7 (272.3-1802.9)	1331.3 (632.9-5020.9)	1731.3 (860.7-7266.1)	3718.0 (760.0-21351.5)
GDPP (ng/ml)	2.8 (1.6–11.2)	4.5 (1.9–7.9)	9.0 (2.7–21.0)	15.3 (4.4-247.9)

Next, we evaluated the correlation between the bone scan index (BSI), an objective, quantitative score calculated using a computer-aided diagnostic system (BONENAVI) for bone scintigraphy, to determine the volume of BM and blood biomarkers. The correlation between GDPP and BSI was the strongest (*r* = 0.66, *p* < 0.01) among all the blood biomarkers examined, including mGDF15 (*r* = 0.43) (Fig. 1D) (*n* = 80). Next, we investigated the changes in GDPP levels (ΔGDPP) in the same CRPC patients with BM over time to determine the clinical utility of GDPP for longitudinal monitoring of BM (Table S5) (*n* = 22). The value of ΔGDPP was significantly associated with ΔBSI (*r* = 0.63, *p* < 0.01), but the changes in PSA levels (ΔPSA) and in all bone turnover markers, except BAP, were not correlated with ΔBSI (Fig. 1E). The representative case showed an increase in serum GDPP levels accompanied by an increase in the standard uptake value (SUV), as diagnosed by [^18^F] PSMA-1007 PET, even though PSA levels remained low 2 years after surgical castration (Fig. 1F). Overall, the changes in GDPP levels generally mirrored the dynamics of the BM burden throughout the clinical course of CRPC in patients with BM.

We also allocated 80 CRPC patients with BM to either the high- or low-GDPP groups and found that the GDPP level was significantly correlated with cancer-specific survival (CSS) (hazard ratio (HR) 11.0, 95% confidence interval (CI) 3.96–30.3, *p* < 0.01) and overall survival (OS) (HR 11.3, 95% CI 4.13-31.0, *p* < 0.01) (Fig. 1G, Fig. S2A). Furthermore, multivariate analysis revealed that GDPP (≥ 15.3 ng/ml) as well as PSA (≥ 17.7 ng/ml) were independent predictors of CSS (HR 7.03, 95% CI 2.30–21.5, *p* < 0.01) and OS (HR 7.26, 95% CI 2.40–21.9, *p* < 0.01) in patients with CRPC (Table 2, Table S6).

**Table 2:** Univariate and multivariate logistic regression analysis of OS in CRPC patients with BM (*n* = 80)

	Univariate analysis			Multivariate analysis		
	HR	95% CI	*P* value	HR	95% CI	*P* value
Age (< 75 vs. ≥75 y)	0.95	0.45–1.99	0.89	-	-	-
Gleason Score (< 8 vs. ≥8)	0.67	0.28–1.60	0.37	-	-	-
PSA (ng/ml) (< 17.7 vs. ≥17.7)	8.30	3.16–21.8	< 0.01	4.68	1.53–14.3	< 0.01
ALP (U/l) (< 186.5 vs. ≥186.5)	1.39	0.65-3.00	0.39	-	-	-
LDH (U/l) (< 210.5 vs. ≥210.5)	1.96	0.92–4.19	0.08	-	-	-
OC (ng/ml) (< 7.8 vs. ≥7.8)	0.67	0.32–1.41	0.29	-	-	-
BAP (µg/l) (< 22.5 vs. ≥22.5)	3.21	1.44–7.18	0.044	1.97	0.81–4.76	0.13
PINP (ng/ml) (< 37.0 vs. ≥37.0)	0.95	0.45-2.00	0.90	-	-	-
TRACP 5b (mIU/dl) (< 237 vs. ≥237)	1.69	0.79–3.62	0.18	-	-	-
GDPP (ng/ml) (< 15.3 vs. ≥15.3)	11.3	4.13-31.0	< 0.01	7.26	2.40–21.9	< 0.01

HR; hazard ratio, CI; confidence interval

To further investigate whether the utility of GDPP as a blood biomarker to detect BM can also be applied to BM with hormone-naive PCa patients, we analyzed HSPC patients with BM (Tables S7, S8, Fig. S2B-S2D). In this population, we also observed that GDPP significantly increased and had the highest diagnostic ability for BM among several blood markers. Interestingly, multivariate analysis also demonstrated that GDPP was significantly associated with the diagnostic accuracy for BM in PCa patients (Table S9) (*n* = 185), while other markers including PSA had no significant association with diagnosis of BM. In addition, there was no significant difference in serum GDPP levels before and after radical prostatectomy (Fig. S2E). The changes in blood GDPP level during systemic therapy were similar to the changes in BSI of a HSPC patient (Fig. S2F).

### Relationship between GDPP levels and aging

Previous studies have suggested that GDF15 is one of the upregulated proteins with aging [39]. To assess the impact of aging, we divided the CRPC patients with BM (*n* = 80) into two groups based on the median age (< 75 years and ≥ 75 years). As a result, there was no significant difference in GDPP values between the two groups (*p* = 0.950, Fig. S2G). We also analyzed the correlation between GDPP levels and the age of all study patients (*n* = 185) and found no clear correlation between GDPP levels and age (*r* = 0.15, Fig. S2H), despite the significant p-value (*p* = 0.038).

### Molecular dynamics of GDPP inside and outside the cell

**Fig. 2:**
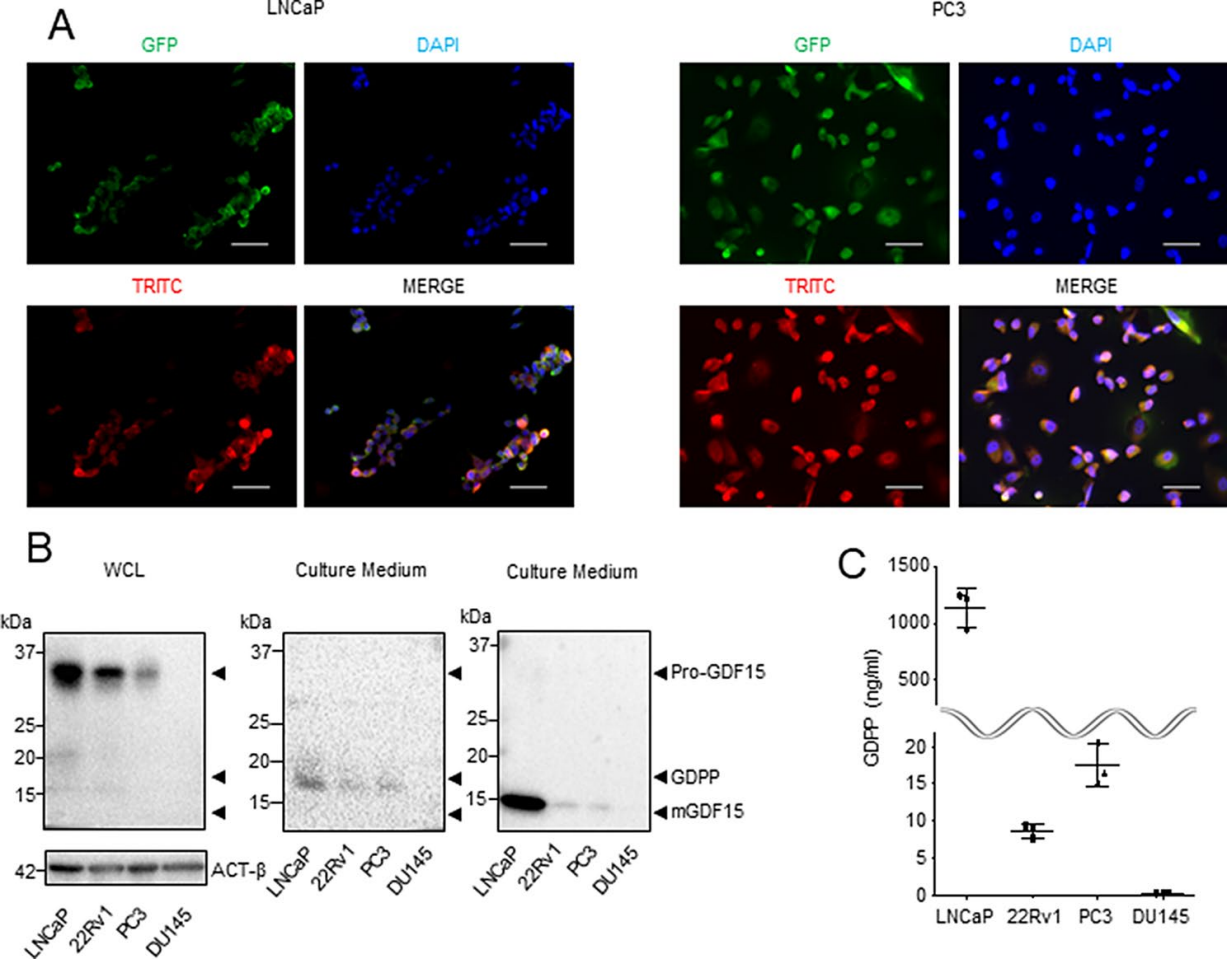
GDPP is cleaved from pro-GDF15 and secreted from PCa. (**A**) Immunofluorescence staining showed the colocalization of GDPP and mGDF15 within LNCaP and PC3 cells. Nuclei are indicated in blue (DAPI); GDPP is indicated in green (GFP), mGDF15 is indicated in red (TRITC). GFP: Green fluorescent protein, TRITC: Tetramethylrhodamine, DAPI: 4’,6-diamidino-2-phenylindole. Scale bar, 50 μm. (**B**) Analysis of GDPP and mGDF15 in PCa cell lines by western blot revealed that GDPP and mGDF15 exist intracellularly as bound pro-GDF15 and extracellularly as separate entities, GDPP and mGDF15, respectively. (**C**) The concentration of GDPP in the culture medium in each of the four PCa cell lines was quantified using ELISA

To examine the subcellular localization of GDPP, we investigated the intracellular spatial relationship between GDPP and mGDF15. Immunofluorescence staining of LNCaP and PC3 cells revealed co-localization of GDPP and mGDF15 in the cytoplasm (Fig. 2A). To verify whether GDPP was truly present as a secreted protein and to determine its levels in various PCa cell lines, we performed western blot analysis and confirmed the exogenous expression of GDPP and mGDF15 in the culture media of LNCaP, 22Rv1, PC3 and DU145 cells, whereas only pro-GDF15 expression was detected in the whole-cell lysate (WCL) of LNCaP, 22Rv1, and PC3 cells (Fig. 2B). These findings suggest the secretion of GDPP into the culture supernatant. In cell lines with high pro-GDF15 expression, both GDPP and mGDF15 were highly expressed in the culture supernatant. When a furin inhibitor was added to the LNCaP cells, neither GDPP nor mGDF15 was secreted into the culture medium; instead, pro-GDF15 accumulated within the treated LNCaP cells (Fig. S3A). Consistent with the findings of the secretome and western blot analyses, LNCaP, 22Rv1, and PC3 cells secreted GDPP into the culture medium, whereas DU145 cells secreted very little GDPP (Fig. 2C). Taken together, these findings indicate that the GDPP peptide is secreted by PCa cells into the extracellular space.

### GDPP promotes prostate cancer cell progression

**Fig. 3:**
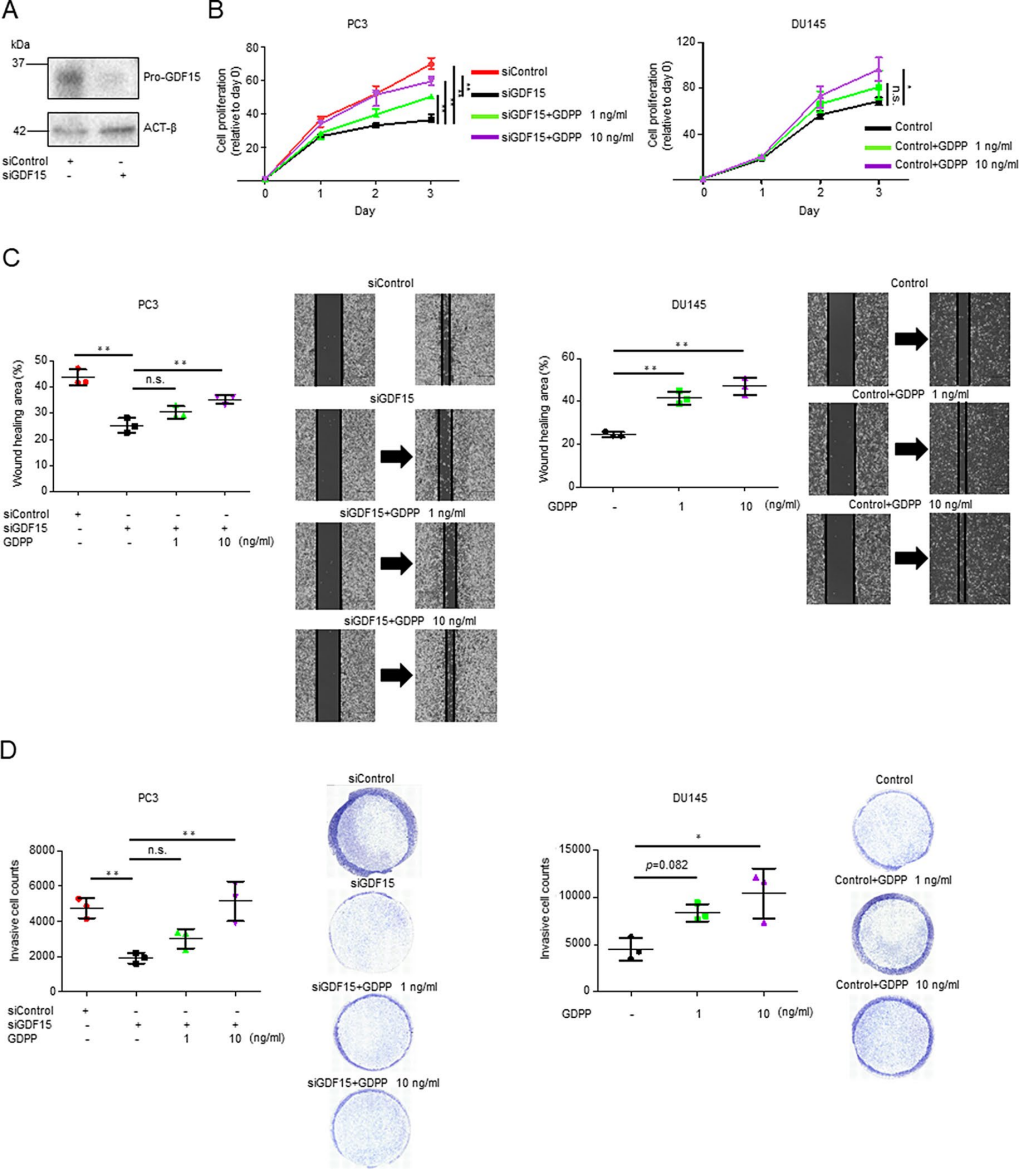
Functional analysis of GDPP in PCa cell lines. (**A**) Western blot analysis of GDPP expression in PC3 transfected with negative control siRNA or siGDF15. (**B**) PC3 transfected with siGDF15 or control siRNA and DU145 seeded with or without rGDPP treatment were incubated and proliferation was examined by MTS cell proliferation assay. (**C**) Scratch wound-healing assays of PC3 transfected with siGDF15 or negative control siRNA and of DU145 treated with or without rGDPP. Scale bar, 500 μm. (**D**) Invasion assays of PC3 transfected with siGDF15 or negative control siRNA and DU145 with or without rGDPP. Data are expressed as the mean ± SD, and statistical analyses were performed using the Tukey‒Kramer method (* *p* < 0.05, ** *p* < 0.01; n.s., not significant)

Next, we focused on the utility of GDPP in CRPC since PSA does not always reflect the BM status of CRPC in clinical settings. To determine why GDPP was more sensitive than PSA as a blood biomarker in patients with CRPC and BM, we sought to elucidate the underlying mechanisms using CRPC cell lines. We explored the function of GDPP in the BM microenvironment through molecular and cellular studies involving RNA silencing. To investigate the biological role of GDPP in various PCa cell processes, we used siRNA to knockdown *GDF15* expression in PC3, which was originally derived from the BM site of a CRPC patient in which *GDF15* is moderately expressed, and DU145, which was derived from the brain metastasis site of a CRPC patient in which *GDF15* exhibited low expression. Transfection of PC3 cells with siGDF15 resulted in a decrease in *GDF15* transcript levels and in the amount of target pro-GDF15 protein (Fig. 3A). These cell lines were then used to evaluate the role of GDPP in various cellular processes associated with cancer progression. To examine the effect of GDPP on PC3 cell proliferation, we treated *GDF15* knockdown cells with recombinant GDPP (rGDPP) (Fig. S3B, S3C) and compared their proliferation levels with those of control cells. The results showed that *GDF15* knockdown significantly decreased cell viability compared to control cells (Fig. 3B). Interestingly, treatment with rGDPP counteracted the effect of *GDF15* knockdown by markedly increasing cell viability in a dose-dependent manner in *GDF15* knockdown cells, suggesting that GDPP promoted the proliferation of PCa cells. Similarly, rGDPP treatment of DU145 cells significantly enhanced their proliferation (Fig. 3B). Furthermore, rGDPP treatment significantly increased the migration and invasion of PC3 and DU145 cells (Fig. 3C, D).

To further evaluate whether rGDPP promotes carcinogenesis even in PCa with androgen-dependent status, we performed functional analysis of GDPP with LNCaP cells which highly produce GDPP into the extracellular space (Fig. 2C). As a result, rGDPP significantly promoted the proliferation and invasion ability of LNCaP cells (Fig. S3D-S3F).

Collectively, these results indicated that GDPP independently promoted PCa cellular processes associated with tumor progression and metastasis, including cell proliferation, migration, and invasion.

### Bone-associated cells secrete GDPP in the bone microenvironment

**Fig. 4:**
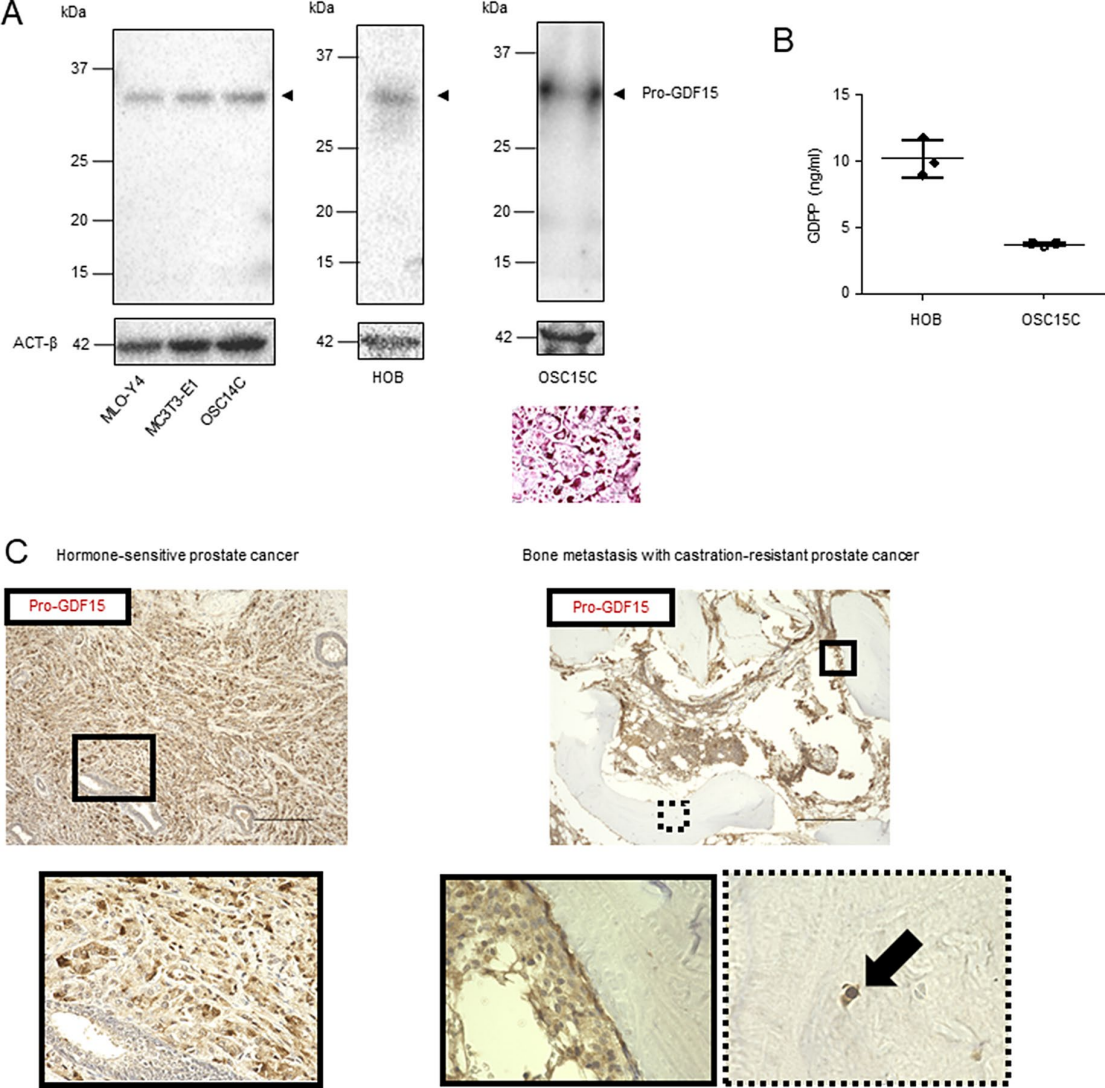
Intracellular and extracellular molecular dynamics of GDPP in HOB and OSC15C. (**A**) Western blot analysis of cell lysates revealed the expression of pro-GDF15 in osteoblasts (MC3T3-E1 and HOB), osteoclasts (OSC14C and OSC15C), and osteocytes (MLO-Y4). Furthermore, the images depict the differentiation of OSC15C cells into mature osteoclasts. Scale bar, 500 μm. (**B**) ELISA-based evaluation of the concentration of GDPP in the culture medium of HOB and OSC15C. (**C**) Immunohistochemical staining of pro-GDF15 in human PCa cells, as well as in osteoblasts, osteoclasts, and osteocytes within the BM of PCa patients. Black arrows indicate osteocytes. Scale bar, 250 μm

Next, because of its higher accuracy in reflecting the extent of BM in CRPC patients than PSA, GDPP was hypothesized to be secreted from osteoblasts or osteoclasts. We also tested whether GDPP is expressed by cells involved in BM. First, we examined the expression levels of pro-GDF15 in HOB and OSC15C cells. Western blotting revealed that pro-GDF15 was expressed in mouse osteoblasts (MC3T3-E1), HOB, mouse osteoclasts (OSC14C), and OSC15C. This result was also observed in mouse osteocytes (MLO-Y4) (Fig. 4A). However, we could not confirm this in human osteocytes because these cells are not commercially available. The results of our ELISA showed that GDPP was secreted by HOB and OSC15C cells into the culture medium (Fig. 4B). Additionally, immunohistochemical analysis of pro-GDF15 in human PCa tissues and the BM of CRPC patients confirmed that pro-GDF15 was expressed not only in PCa tissues but also in bone-related cells, including human osteocytes (Fig. 4C), suggesting that all relevant cell types associated with BM can secrete GDPP in the bone microenvironment. Interestingly, we found a significant increase of the expression in pro-GDF15 in BM compared with that in local prostate tissue or lymph node metastatic tissue in CRPC patients (*p* = 0.0060 and *p* = 0.0148, Fig. S4A-S4D), whereas there was no significant difference of furin expression between those tissues.

To further examine the infiltration of immune cells in BM, we evaluated CD8^+^ and CD80^+^ cells that represent CD8 T cells and macrophages. As shown in Fig S4A-S4C, we found that the expression levels of CD8 and CD80 were extremely low in BM, implying little infiltration of immune cells in BM of PCa patients.

### GDPP promotes bone metabolism by increasing the proliferation and differentiation of human osteoblasts and osteoclasts

**Fig. 5:**
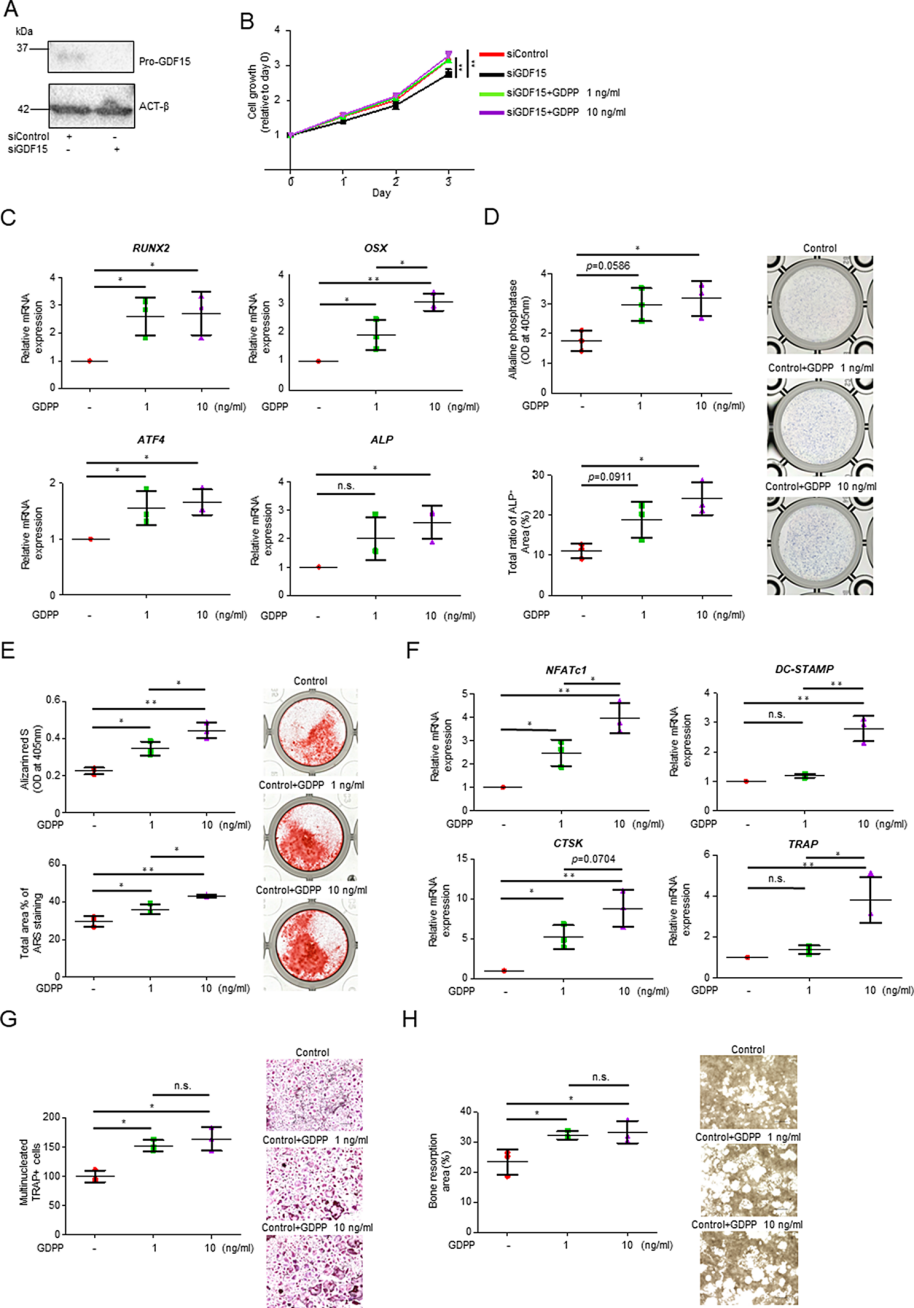
Functional analysis of GDPP in HOB and OSC15C. (**A**) Western blot analysis of the expression of GDPP in HOB. HOB was transfected with negative control siRNA or siGDF15. (**B**) Cell growth curve of HOB lines transfected with siGDF15 or negative control siRNA that were seeded with or without rGDPP, and proliferation was examined by MTS assays. (**C**) Expression analysis of genes related to differentiation of HOB. Total RNA was isolated from HOB treated with or without rGDPP. mRNA expression of *RUNX2*, *OSX*, *ATF4* and *ALP* was evaluated using quantitative real-time PCR analysis. The expression of each gene was normalized to *GAPDH* expression. (**D**) Analysis of ALP activity in HOB. Representative images of ALP staining, OD values and ALP^+^ area percentages of HOB with or without rGDPP are shown. (**E**) Bone mineralization analysis of HOB. Representative images of HOB mineralization detected by Alizarin Red S staining, with OD values and area percentages with or without GDPP are shown. (**F**) Expression analysis of genes related to differentiation of OSC15C. Total RNA was isolated from OSC15C treated with or without rGDPP after differentiation into mature osteoclasts. mRNA expression of *NFATc1*, *DC-STAMP*, *CTSK* and *TRAP* was evaluated using quantitative real-time PCR analysis. The expression of each gene was normalized to *GAPDH* expression. (**G**) TRAP staining analysis of OSC15C. OSC15C was treated with or without rGDPP and stained for TRAP. Scale bar, 500 μm. (**H**) Pit formation analysis of OSC15C. OSC15C was seeded into bone resorption assay plates and treated with or without rGDPP. Representative images of resorption pit formation and the percentage of resorbed area (bright area) were quantified. Data are expressed as the mean ± SD, and statistical analyses were performed using the Tukey‒Kramer method (* *p* < 0.05, ** *p* < 0.01; n.s., not significant). Scale bar, 500 μm

To investigate the biological function of GDPP in the bone microenvironment, we evaluated whether GDPP influenced the proliferation and viability of osteoblasts. First, we confirmed that siRNA successfully reduced the amount of GDPP expressed in HOB cells (Fig. 5A), which significantly decreased their proliferation (Fig. 5B). Notably, the addition of rGDPP increased the viability of HOB treated with siGDF15 in a dose-dependent manner (Fig. 5B). We examined the transcriptional regulation in HOB cells treated with rGDPP. Gene expression analysis showed that rGDPP treatment increased the transcript levels of osteoblast-related genes essential for bone formation, including *RUNX2*, *OSX*, *ATF4*, and *ALP* (Fig. 5C, Fig. S5A). In addition, rGDPP treatment increased ALP activity, which is an indicator of osteoblastic differentiation and bone mineralization (Fig. 5D and E). These findings suggest that GDPP may enhance the osteogenic potential by increasing the expression of transcription factors in human osteoblasts and promoting their differentiation.

To investigate whether GDPP influences osteoclast proliferation and viability, we evaluated the effect of GDPP on OSC15C cells, which are related to bone absorption in the bone microenvironment. The addition of rGDPP to OSC15C cells resulted in increased expression levels of differentiation-related genes such as *NFATc1*, *DC-STAMP*, *CTSK*, and *TRAP* (Fig. 5F, Fig. S5B). We also found that the addition of rGDPP to OSC15C cells led to a significant increase in mature osteoclasts (Fig. 5G) and bone resorption potential (Fig. 5H). Collectively, these findings suggested that GDPP enhanced bone metabolism by upregulating the expression of osteogenic and osteoclastic factors in the bone microenvironment.

### GDPP promotes bone metastasis of CRPC in vivo

**Fig. 6:**
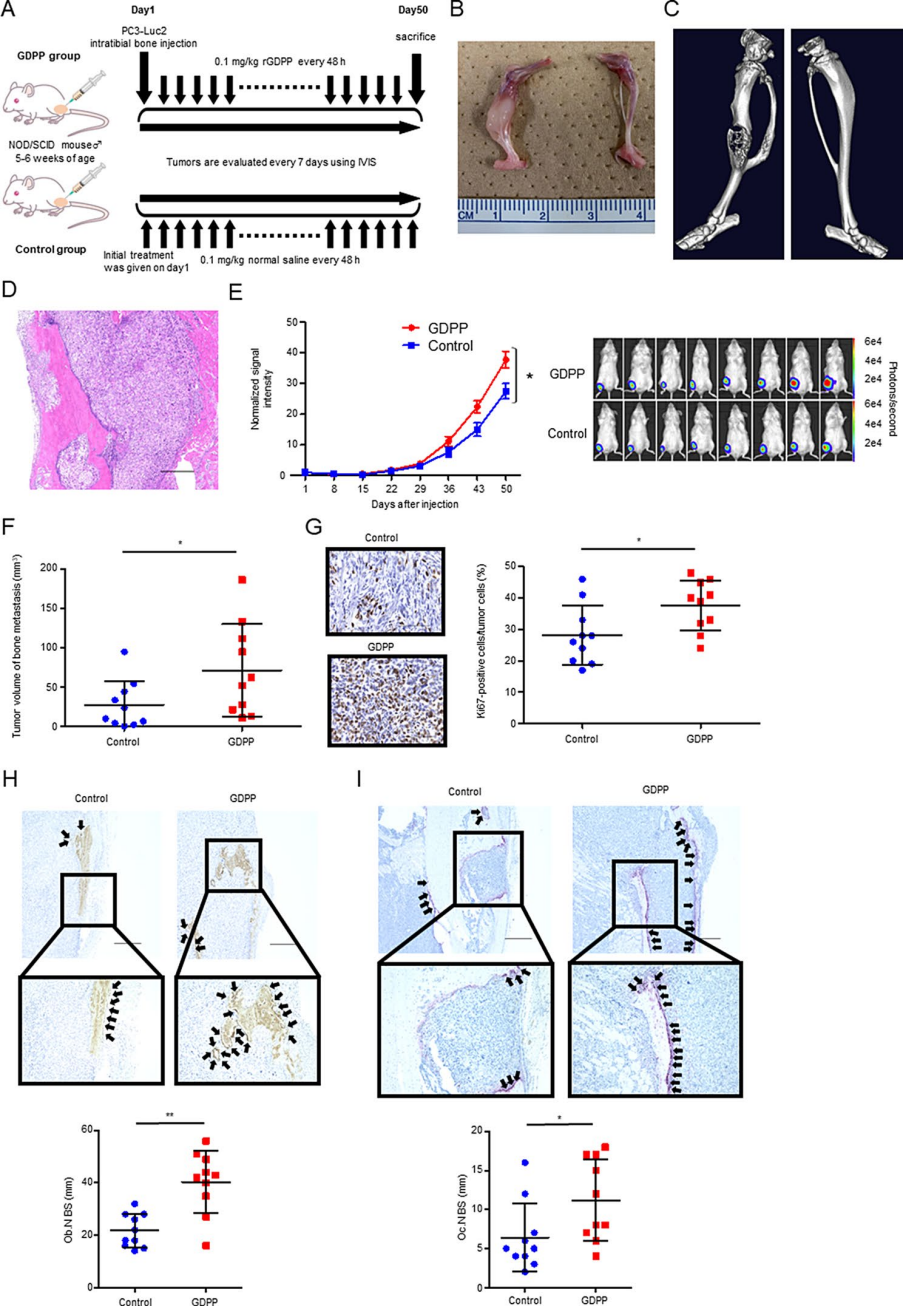
Functional analysis of GDPP in a xenograft model of human PCa cells within the bone microenvironment. (**A**) Schema of the experimental procedure. PC3-Luc2 cells were directly injected into the tibia of male NOD/SCID mice. The mice were subsequently subcutaneously administered rGDPP (*n* = 10), while the control group received saline injections (*n* = 10). Weekly imaging using an IVIS was performed. (**B**) Macroscopic image of a tibial bone tumor 50 days following the injection of PC3-Luc2 cells. (**C**) µCT and 3D modeling images demonstrated that the tumor cells had invaded the tibial bone. Scale bar, 500 μm. (**D**) Histological staining using hematoxylin and eosin revealed the infiltration of tumor cells into the tibial bone. Scale bar, 500 μm. (**E**) Weekly bioluminescence imaging captured changes in the tumor growth pattern of PC3-Luc2 cells within the tibia over time (*n* = 20). (**F**) The comparison of bone tumor volume 50 days after intratibial injection (*n* = 20). (**G**) Representative images of immunohistochemical staining for Ki67 in bone tumor and quantitative comparison of Ki67- positive cells (control: *n* = 10, GDPP: *n* = 10). Scale bar, 100 μm. (**H**) The number of OC-positive cells was quantified per unit trabecular bone surface (*n* = 20). Black arrows indicate osteoblasts. Scale bar, 500 μm. (**I**) The number of TRAP-positive cells was quantified per unit trabecular bone surface (*n* = 20). Black arrows indicate osteoclasts. Scale bar, 500 μm. Data are expressed as the mean ± SD, and statistical analyses were performed using a Mann–Whitney U test (* *p* < 0.05, ** *p* < 0.01)

These results prompted us to investigate whether GDPP administration promotes BM development in preclinical models (Fig. 6A). In our established models, we first confirmed bone invasion by cancer cells into the bone substrate (Fig. 6B-D, Fig. S6). Consistent with the in vitro results (*n* = 20), rGDPP administration increased the development of CRPC tumor in the BM compared to that in control mice, as evidenced by the significant increase of ROI, tumor volume, and Ki67-positive cells (*p* = 0.0343, *p* = 0.0412, and *p* = 0.0445, respectively, Fig. 6E-G). We also confirmed that the number of osteoblasts and osteoclasts was significantly higher in the tumors of the rGDPP treatment group (*n* = 10) than in those of the control group (*n* = 10), suggesting that GDPP enhances the proliferation of osteoblasts and osteoclasts in the bone microenvironment and may augments the tumor microenvironment of BM (Fig. 6H, I).

## Discussion

BM can occur in various types of cancers and significantly reduces the quality of life of patients by causing skeleton-related events leading to serious immobility [40]. CRPC is an advanced form of PCa that develops due to disease progression following surgical or chemical castration, and approximately 80–90% of patients with CRPC develop BM, which significantly affects clinical prognosis [41]. In general, for the diagnosis of PCa, whole-body CT and bone scintigraphy are performed to assess metastasis in organs, particularly the bone. However, bone scintigraphy does not lead to a definitive diagnosis because of some limitations, including limited availability of facilities, exposure to radiation, and a high false-positivity rate [7]. Guidelines for BM diagnosis currently do not provide information on effective imaging examinations or blood biomarkers, making early diagnosis challenging [6, 42]. Therefore, we aimed to identify a convenient and accurate diagnostic biomarker for BM monitoring.

NEPC is a histological variant of PCa characterized by aggressiveness and poor clinical outcomes and occurs in approximately 20% of patients with mCRPC. In general, PSA levels do not reflect disease status in NEPC because NEPC-derived cancer cells scarcely produce PSA, leading to a difficult challenge in understanding the disease [11]. Hence, in this study, we focused on GDF15, which has been reported to be expressed in NEPC [37, 38], with the aim of investigating its potential as a useful biomarker not only in CRPC patients but also in NEPC patients who require the precise assessment of BM. In this study, we demonstrated several novel findings that identified GDPP as a novel biomarker for the diagnosis and monitoring of BM in patients with CRPC.

First, we found that a unique propeptide, GDPP, was secreted by PCa cells, which is also secreted by bone-associated cells: osteoblasts and osteoclasts (Figs. 2B and C and 4A and B). GDPP is normally expressed intracellularly as pre-pro-GDF15, which is then cleaved into mGDF15 and GDPP by furin. So far, previous studies have shown that mGDF15 is secreted extracellularly and may promote cancer progression [21, 43]. In contrast, similar to the C-peptide, which is a precursor of proinsulin, the propeptide domain of a protein is sometimes considered functional, which prompted us to investigate the detailed functions of GDPP [44, 45]. As expected, our study revealed that secreted GDPP promotes PCa carcinogenesis and the proliferation of bone-associated cells, leading to the development of BM (Figs. 3B-D, 5B-H and 6E-I).

The high incidence of BM in PCa is believed to involve the development of a bone microenvironment that supports the growth of PCa cells, as indicated by the seed and soil theory [46]. For instance, growth factors such as the TGF-β family are released and activated in response to bone tissue degradation and various changes in the bone microenvironment. In this context, CXCR4 has been reported to be a therapeutic target because TGF-β signaling induces acetylation of the transcription factor KLF5 in PCa with BM, which activates CXCR4, leading to osteoclastogenesis and BM [47, 48]. Our results also highlighted the pivotal role of GDPP in bone microenvironment including osteoblastic and osteolytic BM, which may be a candidate therapeutic target for patients with CRPC.

We also found a significant increase in GDPP levels in CRPC patients with BM compared to those in CRPC patients with visceral metastases or locally advanced disease (Table 1; Fig. 1A-C). GDPP is considered a superior biomarker compared to tumor and bone turnover markers, all of which are conventionally used for the diagnosis of BM, because GDPP has synergistic effects on PCa cells, osteoblasts, and osteoclasts during the progression of BM. Indeed, there was no significant difference in the GDPP levels between patients with localized PCa and healthy donors (Fig. 1A). Given that there was no influence on GDPP levels after radical prostatectomy (*n* = 179) (Table S10, Fig. S2E), we speculated that the GDPP value would drastically increase once BM began. In fact, GDPP levels were significantly correlated with BM volume not only in CRPC patients (Fig. 1D, E) and HSPC patients receiving systemic therapy (Fig. S2C). These findings suggest that GDPP, which is not a traditional osteogenic marker, may perceptively diagnose BM and reduce radiological imaging tests, leading to an early diagnosis of oligometastases and thereby earlier intervention in PCa patients (Fig. 1F).

**Fig. 7:**
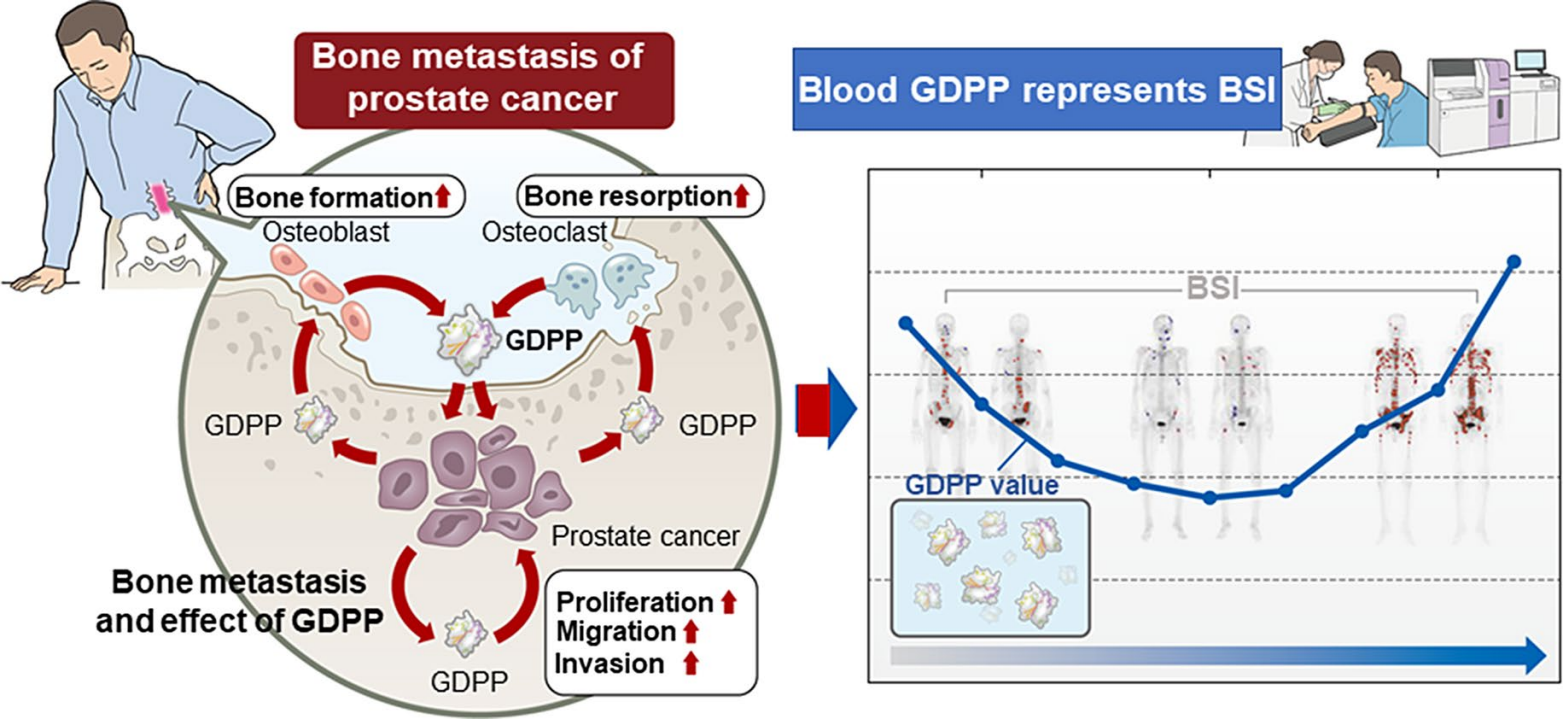
Graphical abstract showing the role of GDPP in promoting bone metastasis in CRPC patients. In CRPC patients, GDPP augments the tumor microenvironment of BM and is an accurate biomarker for BM. The illustration on the left shows the schema in which PCa cells, osteoblasts, and osteoclasts secrete GDPP, PCa progresses, and osteoblasts and osteoclasts also proliferate, each of which exacerbates BM. The illustration on the right shows that blood GDPP levels in CRPC patients with BM reflect BSI accurately and are a very useful biomarker

Furthermore, we found that the change in GDPP levels reflected the clinical course of BM volume, as evidenced by the change in BSI or SUV in imaging tests (Fig. 1F, Fig. S2F). In addition, multivariate analysis revealed that GDPP was an independent poor prognostic factor for CSS and OS in patients with CRPC and BM (Table 2, Table S6). Considering that PSA levels often do not serve as an indicator of disease status in patients with CRPC and NEPC, we believe that GDPP measurements may be useful for disease monitoring in daily practice (Fig. 7). Future studies are required to validate our results for diagnosing BM at earlier stages using GDPP measurements before imaging tests during progression from HSPC to the lethal CRPC status.

This study has several limitations. First, the GDPP receptor was not identified and the detailed pathway underlying the effects of GDPP has not yet been elucidated. We further need to evaluate the interaction between cancer and bone-associated cells after identifying GDPP receptor. Second, PSMA-PET is not covered by insurance in Japan, making it difficult to assess tumor volume using PSMA-PET in routine clinical practice. To investigate the clinical utility of GDPP more effectively, our future work will aim to prospectively examine patients with CRPC using GDPP, BSI, and PSMA PET by conducting clinical trials to determine whether GDPP is prognostically elevated at the time of CRPC diagnosis and whether it is useful for the diagnosis of bone oligometastases, contributing to early therapeutic intervention and improved prognosis. Third, we observed an unclear association between plasma GDPP levels and age, although mGDF15 is one of the upregulated proteins during aging [39]. Although a large amount of GDPP in BM may alleviate the influence of aging in PCa patients, further investigations are needed to elucidate the process about secretion and degradation of GDPP.

In conclusion, we demonstrated that the GDF15 propeptide, GDPP, is secreted from PCa cells, osteoblasts, and osteoclasts into the blood circulation of patients, promoting the BM of PCa by possible alteration of the bone microenvironment. Therefore, we believe that GDPP is a novel clinically useful blood biomarker that reduces the need for imaging studies and is a new therapeutic target in patients with CRPC and BM.

## Electronic supplementary material

Below is the link to the electronic supplementary material.

Supplementary Material 1

Supplementary Material 2

Supplementary Material 3

Supplementary Material 4

Supplementary Material 5

Supplementary Material 6

Supplementary Material 7

Supplementary Material 8

## Data Availability

No datasets were generated or analysed during the current study.

## Abbreviations

BM: Bone metastasis
CRPC: Castration-resistant prostate cancer
PCa: Prostate cancer
GDF15: Growth differentiation factor 15
GDPP: Growth differentiation factor 15 propeptide
AUC: Area under the curve
PSA: Prostate-specific antigen
mHSPC: Metastatic hormone-sensitive prostate cancer
ALP: Alkaline phosphatase
TGF-β: Transforming growth factorβ
mGDF15: Mature growth differentiation factor 15
BAP: Bone alkaline phosphatase
TRACP 5b: Tartrate-resistant acid phosphatase 5b
LDH: Lactate dehydrogenase
OC: Osteocalcin
PINP: Procollagen I N-terminal propeptide
BSI: Bone scan index
EDTA: Tris-ethylenediaminetetraacetic acid
PBS: phosphate-buffered saline
IVIS: In vivo imaging system
NEPC: neuroendocrine prostate cancer
3D: Three-dimensional
TCGA: The cancer genome atlas
WCL: Whole-cell lysate
ROC: Receiver operating characteristic
SUV: Standard uptake value
CSS: Cancer-specific survival
OS: Overall survival
rGDPP: Recombinant growth differentiation factor 15 propeptide
ATCC: American type culture collection
FBS: Fetal bovine serum
HR: Hazard ratio
CI: Confidence interval
