# Supplementary material for: GDF15 propeptide promotes bone metastasis of castration-resistant prostate cancer by augmenting the bone microenvironment
Source: Biomark Res. 2024 Nov 25;12:147. doi: 10.1186/s40364-024-00695-6 (PMC11590406; doi:10.1186/s40364-024-00695-6)
Supplement: Supplementary file 1 — Supplementary Material 1 [file 40364_2024_695_MOESM1_ESM.docx]

Supplementary Materials for

**GDF15 propeptide promotes bone metastasis of castration-resistant prostate cancer** **by augmenting the bone microenvironment**

Gaku Yamamichi *et al*.

Corresponding author: Taigo Kato, kato@uro.med.osaka-u.ac.jp

**The Word file includes:**

Figures. S1 to S6

Tables S1 to S10

**
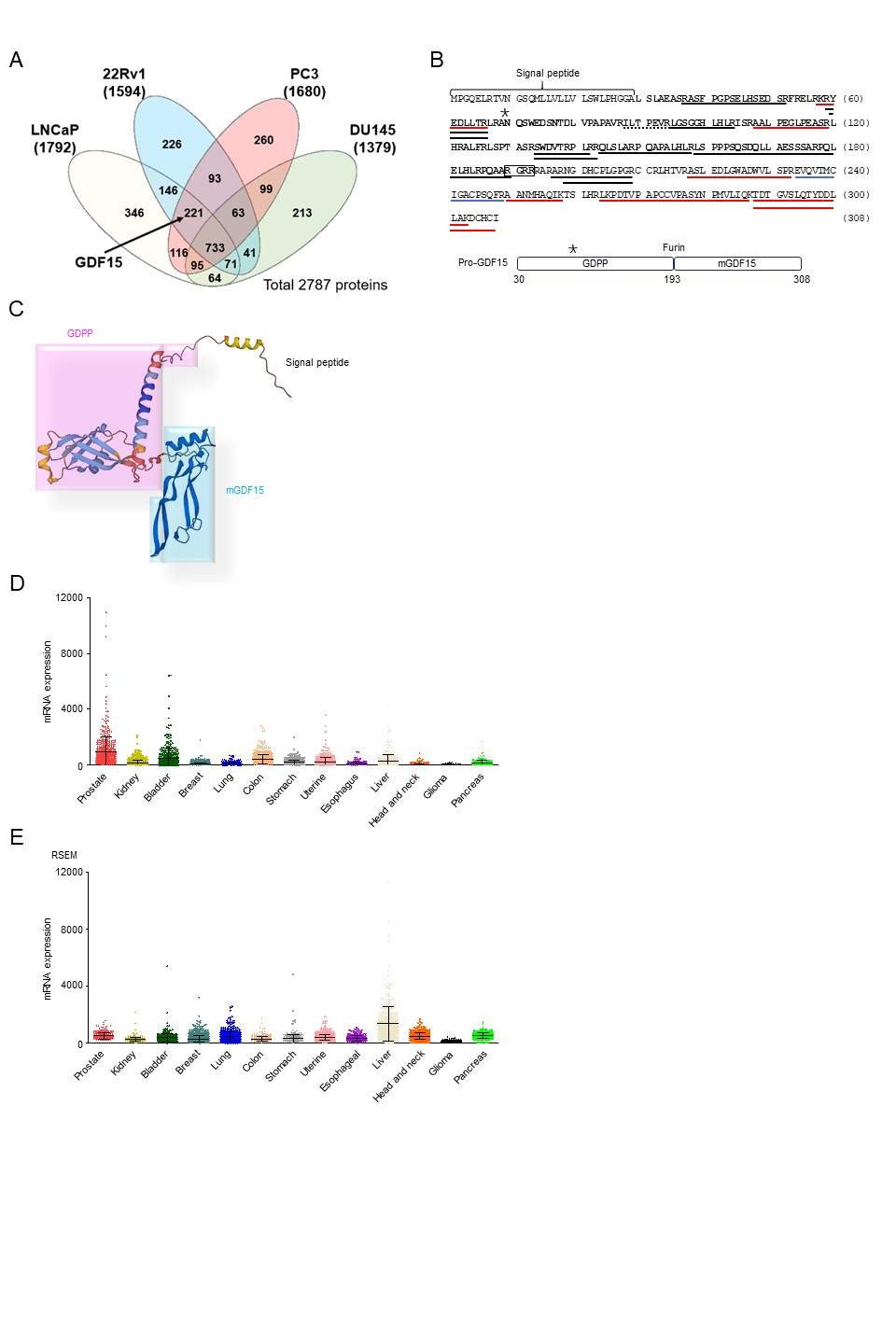
Supplementary Figures**

**Fig. S1 Secretome analysis of PCa cell lines and expression of *GDF15* and *furin* in public data.**

(**A**) Of the culture medium from LNCaP, 22Rv1, PC3, and DU145 cell lines, a total of 2,787 proteins were identified through secretome analysis. (**B**) Peptides from the pro-GDF15 precursor annotated in the secretome analysis of PCa cell lines. Solid red lines indicate detection in three PCa cell lines (LNCaP, 22Rv1 and PC3). Solid blue lines indicate detection in two PCa cell lines (LNCaP and 22Rv1). Solid black lines indicate detection in one PCa cell line (LNCaP). Dotted black lines indicate detection in one PCa cell line (22Rv1). *: N-glycan binding site, □: furin protease consensus sequence (RXXR). The amino acid sequence before the black box is GDPP, and the amino acid sequence after the black box is the amino acid sequence of mGDF15. (**C**) The AlphaFold2 program was used to predict the 3D structure of mGDF15. The signal peptide, GDPP, and mGDF15 are connected to form pre-pro-GDF15. (**D**) RNA-seq data for *GDF15* obtained from TCGA database. (**E**) RNA-seq data for *Furin* from obtained from TCGA database.


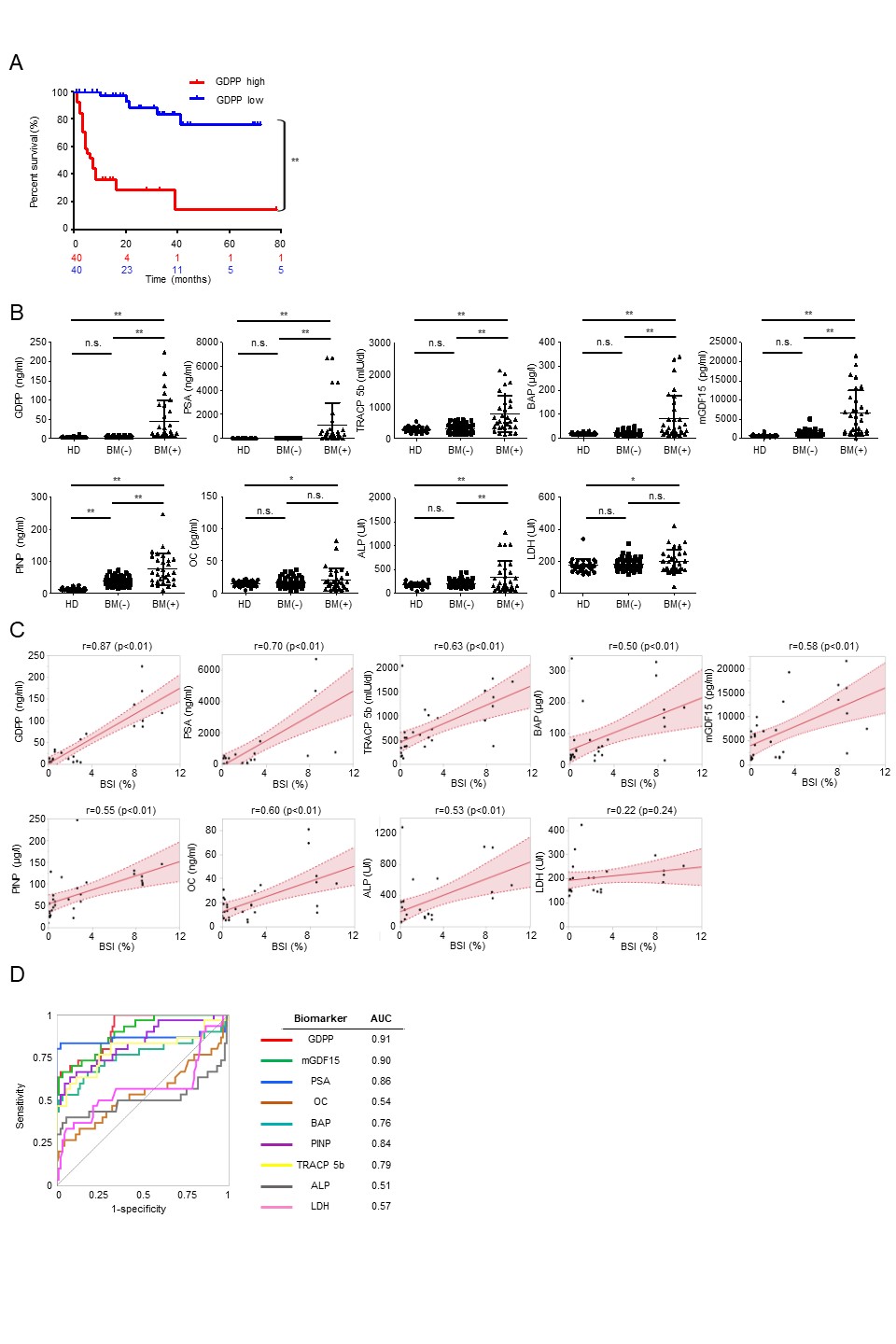


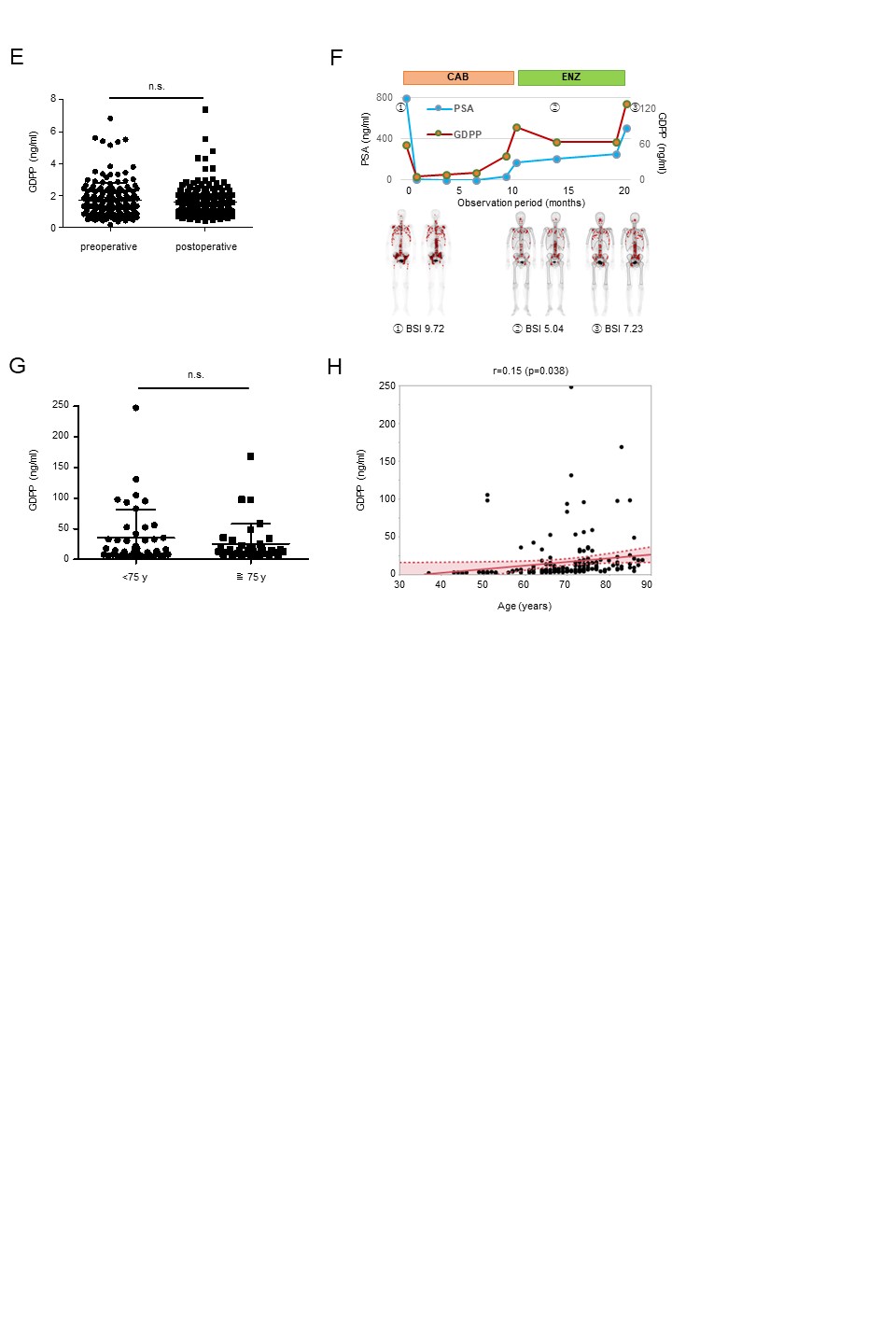
**Fig. S2 Real-world data on GDPP in patients with prostate cancer**

(**A**) Kaplan‒Meier analysis of CSS for CRPC patients with BM stratified by GDPP value, with statistical analyses performed using the log-rank test (** *p* < 0.01). (**B**) Comparison of levels of various blood biomarkers between healthy donors (HD, n=30) and hormone-sensitive prostate cancer (HSPC) patients with or without BM (n=60 and 30, respectively). Patients with BM had elevated levels of GDPP, PSA, TRACP 5b, BAP, mGDF15, PⅠNP and ALP. Data are presented as the mean ± SD, and statistical analyses were performed using the Tukey‒Kramer method (* p < 0.05, ** p < 0.01; n.s., not significant). (**C**) The relationship between the BSI and GDPP, PSA, TRACP 5b, BAP, mGDF15, PINP, OC, ALP, and LDH levels in HSPC patients with BM showed that GDPP correlated most strongly with the BSI (n=30). Statistical analyses were performed using Spearman's rank correlation coefficient. (**D**) ROC analysis showing the AUC to compare the diagnostic performance of various blood biomarkers for BM in HSPC patients (n=30). (**E**) Changes in serum GDPP levels before and within 2 weeks after radical prostatectomy in patients with localized PCa (n=179) (n.s., not significant). (**F**) This panel shows a representative patient treatment course which included systemic treatments such as CAB and ENZ. Plasma GDPP and serum PSA levels show similar dynamics to BSI. CAB: combined androgen blockade therapy, ENZ: enzalutamide, BSI: bone scan index. (**G**) Comparison of blood GDPP levels between younger and older CRPC patients with BM, divided into two groups by median age of 75 years (n=37 and 43, respectively) using the wilcoxon signed-rank test (n.s., not significant). Data are expressed as the mean ± SD. (**H**) Correlation between age and blood GDPP levels (n=185). Statistical analyses were performed using Spearman's rank correlation coefficient.


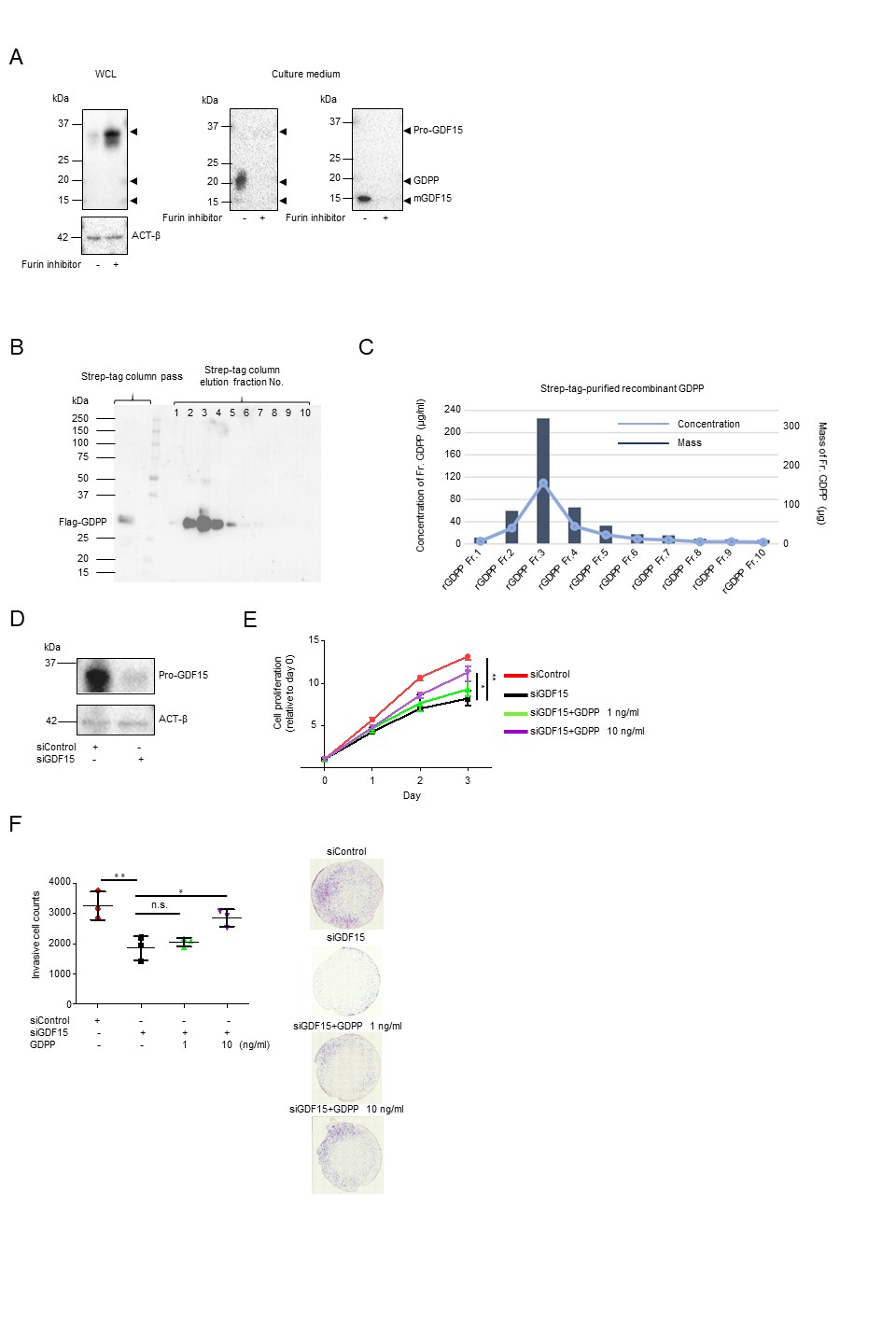


**Fig. S3 Molecular dynamics of GDPP inside and outside the cell and the function of recombinant GDPP to LNCaP**

(**A**) Western blot analysis of the cell lysates and supernatant after furin inhibitor addition to LNCaP. (**B**) Western blot showed the expression of GDPP in the medium of Expi293 cells overexpressing GDPP, and (**C**) ELISA showed the concentration of the recombinant GDPP which collected and purified from this medium. (**D**) Western blot analysis of GDPP expression in LNCaP transfected with negative control siRNA or siGDF15. (**E**) LNCaP transfected with siGDF15 or control siRNA seeded with or without rGDPP treatment were incubated and proliferation was examined by MTS cell proliferation assay. (**F**) Invasion assays of LNCaP transfected with siGDF15 or negative control siRNA with or without rGDPP. Data are expressed as the mean ± SD, and statistical analyses were performed using the Tukey‒Kramer method (* *p* < 0.05, ** *p* < 0.01; n.s., not significant).


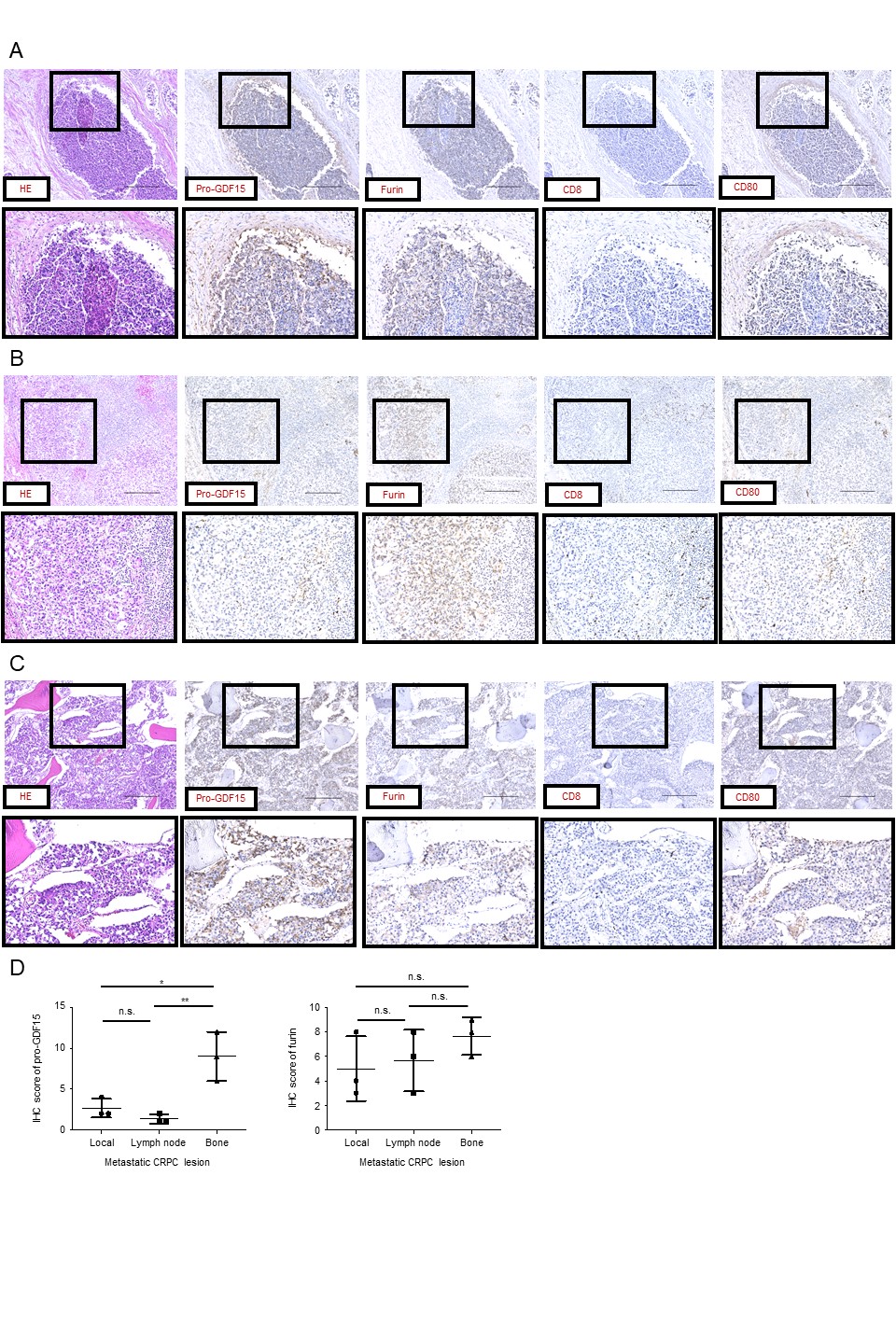


**Fig. S4 Immunohistochemical evaluation using three mCRPC patients with primary tumor, lymph node metastasis, and bone metastatic tissue samples.**

(**A**) Representative images of pro-GDF15, furin, CD8, and CD80 expression in primary tumor, (**B**) lymph node metastasis, and (**C**) BM in the same CRPC patient. Scale bars, 300 μm. (**D**) Quantitative comparison of pro-GDF15 and furin expressions by metastatic site in CRPC patients using immunohistochemistry (n=3). (* *p* < 0.05, ** *p* < 0.01; n.s., not significant).


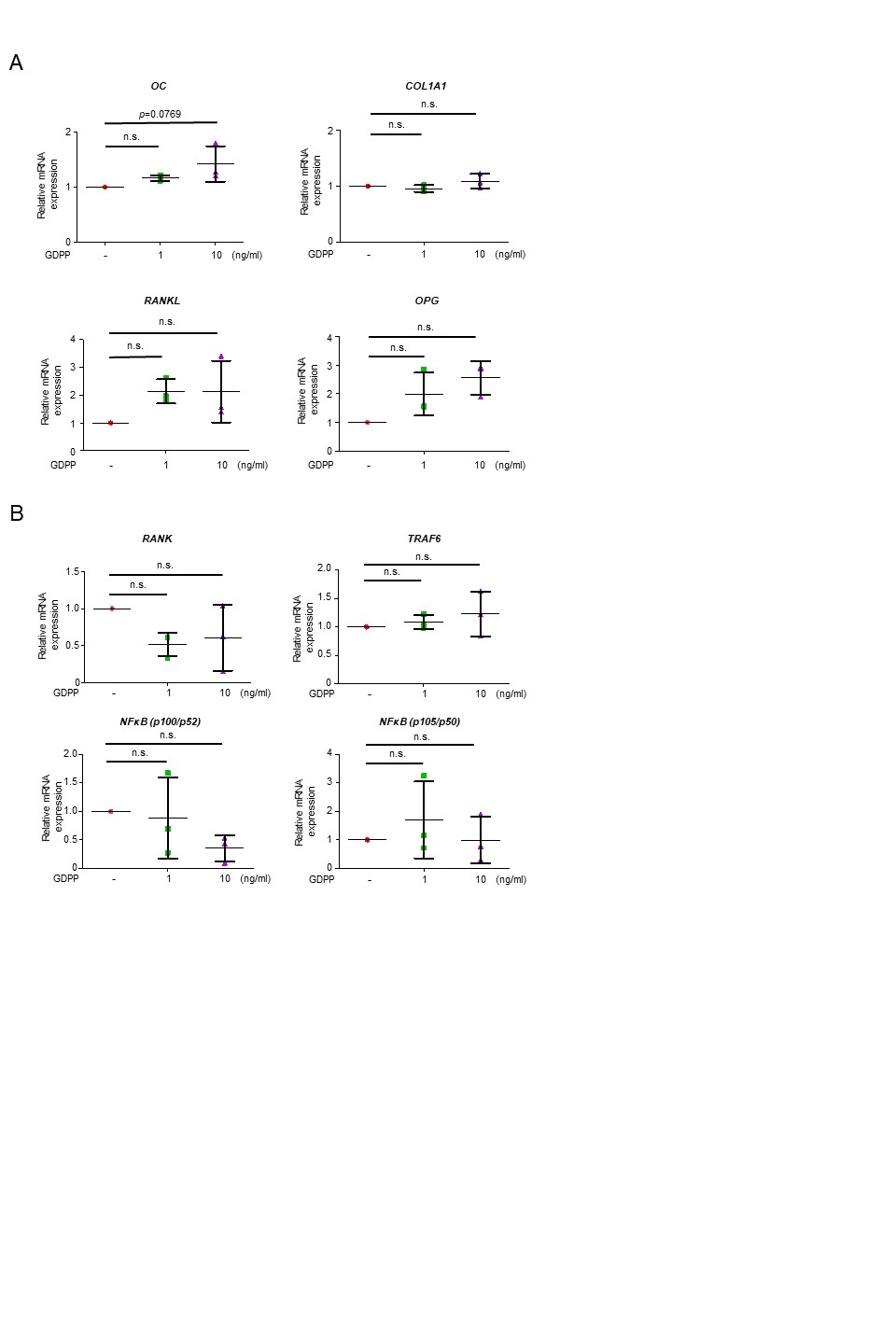


**Fig. S5 Functional analysis of changes in transcription in HOB and OSC15C with GDPP treatment**

(**A**) Total RNA isolated from HOB treated with or without rGDPP. mRNA expression of *OC*, *COL1A1*, *RANKL* and *OPG* was evaluated using quantitative real-time PCR analysis. The expression of each gene was normalized to *GAPDH* expression. (**B**) Total RNA isolated from OSC15C treated with or without rGDPP after differentiation into mature osteoclasts. mRNA expression of *RANK*, *TRAF6*, *NFκB (p100/p52)* and *NFκB (p105/p50)* was evaluated using quantitative real-time PCR analysis. The expression of each gene was normalized to *GAPDH* expression. Data are presented as the mean ± SD, and statistical analyses were performed using the Tukey‒Kramer method (n.s., not significant). The statistical results of three independent experiments are summarized.


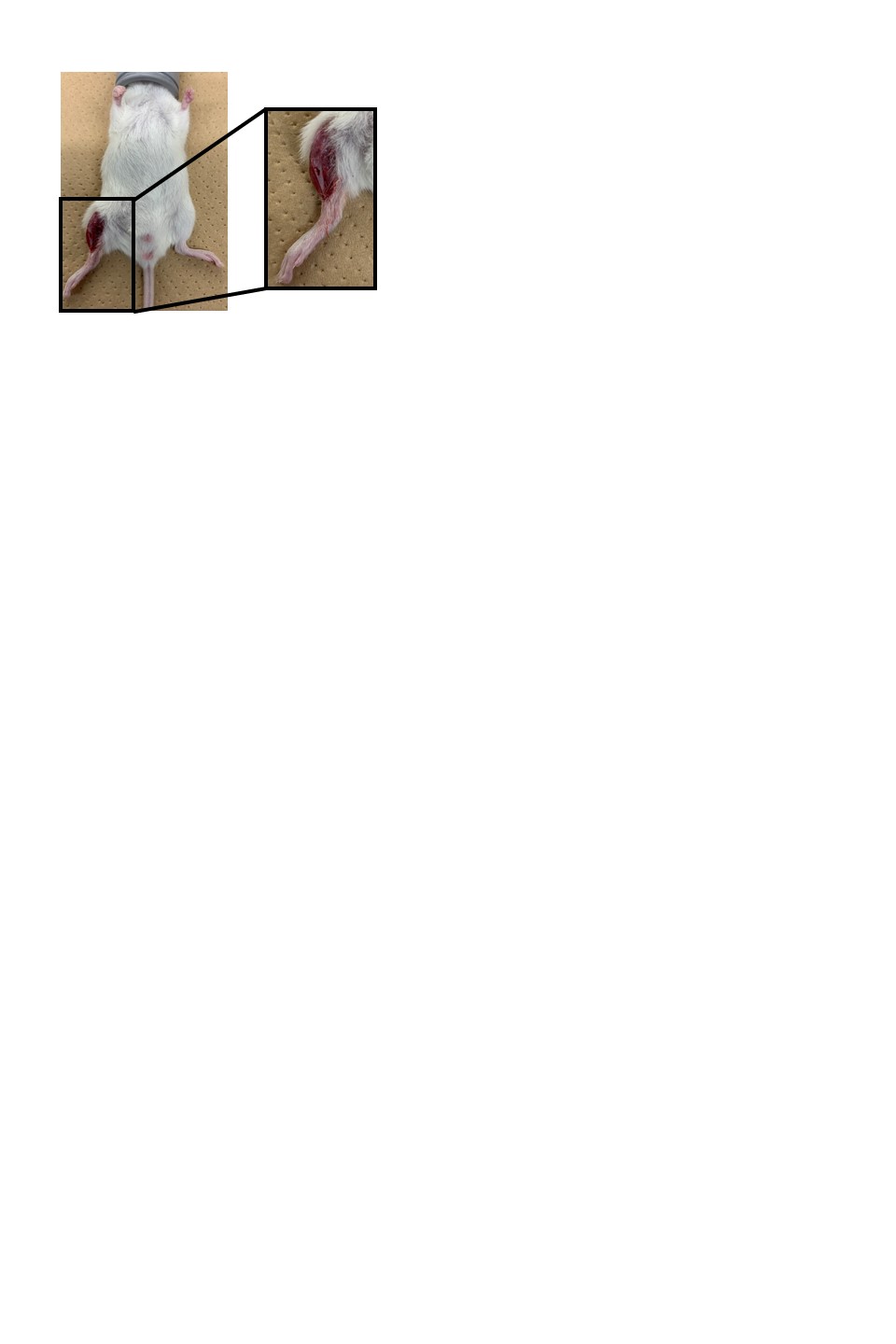
**Fig. S6 A xenograft bone metastasis model created by pin-hole drilling in the tibia**

A pinhole was created in the mouse tibia with a 22G needle for injection of PC3-Luc2 cells.

**Supplementary Tables**

**Table S1.** The primers used for quantitative real-time PCR

| RUNX2*-*F | 5'-TGGTTACTGTCATGGCGGGTA-3' |
| --- | --- |
| RUNX2*-*R | 5'-TCTCAGATCGTTGAACCTTGCTA-3' |
| OSX*-*F | 5'-CCTCTGCGGGACTCAACAAC-3' |
| OSX*-*R | 5'-AGCCCATTAGTGCTTGTAAAGG-3' |
| ATF4*-*F | 5'-ATGACCGAAATGAGCTTCCTG-3' |
| ATF4*-*R | 5'-GCTGGAGAACCCATGAGGT-3' |
| ALP*-*F | 5'-ACCACCACGAGAGTGAACCA-3' |
| ALP*-*R | 5'-CGTTGTCTGAGTACCAGTCCC-3' |
| *OC-*F | 5'-CACTCCTCGCCCTATTGGC-3' |
| *OC-*R | 5'-CCCTCCTGCTTGGACACAAAG-3' |
| COL1A1*-*F | 5'-GTTGCTGCTTGCAGTAACCTT-3' |
| COL1A1*-*R | 5'-AGGGCCAAGTCCAACTCCTT-3' |
| RANKL*-*F | 5'-TCGATGGCTCATGGTTAGATC-3' |
| RANKL*-*R | 5'-GGAACCAGATGGGATGTCGG-3' |
| OPG*-*F | 5'-GCGCTCGTGTTTCTGGACA-3' |
| OPG*-*R | 5'-AGTATAGACACTCGTCACTGGTG-3' |
| TRAP*-*F | 5'-GACTGTGCAGATCCTGGGTG-3' |
| TRAP*-*R | 5'-GGTCAGAGAATACGTCCTCAAAG-3' |
| CTSK*-*F | 5'-ACACCCACTGGGAGCTATG-3' |
| CTSK*-*R | 5'-GACAGGGGTACTTTGAGTCCA-3' |
| NFATc1*-*F | 5'-CACCGCATCACAGGGAAGAC-3' |
| NFATc1*-*R | 5'-GCACAGTCAATGACGGCTC-3' |
| RANK*-*F | 5'-AGAAGACGGTGCTGGAGTCT-3' |
| RANK*-*R | 5'-TAGGAGCAGTGAACCAGTCG-3' |
| TRAF6*-*F | 5'-ATGCGGCCATAGGTTCTGC-3' |
| TRAF6*-*R | 5'-TCCTCAAGATGTCTCAGTTCCAT-3' |
| DC-STAMP*-*F | 5'-CGCTGCCTCCTGGATTATCAC-3' |
| DC-STAMP*-*R | 5'-AAGCTCTTTGCCCTTAGGTTG-3' |
| NF-kB(p105/p50)*-*F | 5'-GAAATTCCTGATCCAGACAAAAAC-3' |
| NF-kB(p105/p50)*-*R | 5'-ATCACTTCAATGGCCTCTGTGTAG-3' |
| NF-kB(p100/p52)*-*F | 5'-CTGGTGGACACATACAGGAAGAC-3' |
| NF-kB(p100/p52)*-*R | 5'-ATAGGCACTGTCTTCTTTCACCTC-3' |
| *GAPDH-*F | 5'-TGTGTCCGTCGTGGATCTGA-3' |
| *GAPDH-*R | 5'-TTGCTGTTGAAGTCGCAGGAG-3' |

**Table S2.** Summary of the data from secretome analysis

|  | | LNCaP | 22Rv1 | PC3 | DU145 |
| --- | --- | --- | --- | --- | --- |
| #, Identified proteins (all) | | 2787 proteins | | | |
| #, Identified peptides (all) | | 17798 peptides | | | |
| #, Identified proteins | | 1792 | 1594 | 1680 | 1379 |
| #, Identified peptides | | 9506 | 8486 | 9834 | 6994 |
| #, Unique peptide | GDF15 (Q99988) | 18 | 9 | 7 | 0 |
|  | PSA  (P07288-1) | 14 | 3 | 1 | 1 |
|  | PAP  (P15309-1) | 3 | 1 | 0 | 0 |

PSA: prostate-specific antigen

PAP: prostatic acid phosphatase

**Table S3.** Patient characteristics and blood biomarkers of the two cohorts. All continuous data are presented with median value and range.

| Cohort 1 | | | | |
| --- | --- | --- | --- | --- |
|  | Healthy donors  n=15 | Patients with local PCa  n=30 | Patients with mCRPC (BM-)  n=8 | Patients with mCRPC (BM+)  n=40 |
| Age (years) | 64  (37-72) | 70  (53-79) | 74  (59-86) | 74  (51-88) |
| Gleason Score  <8  ≥8 | - | 26  4 | 3  5 | 5  35 |
| pT stage pT2  pT3 | - | 23  7 | - | - |
| PSA (ng/ml) | 0.74  (0-3.2) | 6.1  (2.6-45.5) | 4.8  (0-110.8) | 37.0  (0.1-4782) |
| ALP (U/l) | 183  (101-292) | 187  (107-321) | 166  (60-334) | 173  (45-4987) |
| LDH (U/l) | 185  (129-213) | 175  (120-312) | 191  (175-280) | 231  (115-853) |
| OC (ng/ml) | 14.8  (10.5-21.4) | 14.7  (3.6-35.8) | 13.6  (9.0-36.8) | 7.1  (1.2-109.5) |
| BAP (μg/l) | 21.0  (14.1-30.7) | 22.2  (10.5-41.7) | 25.3  (3.7-45.3) | 25.3  (10.2-332.4) |
| PⅠNP (ng/ml) | 11.9  (6.5-22.4) | 42.2  (14.8-63.4) | 57.1  (40.2-169.4) | 39.9  (5.2-280.9) |
| TRACP 5b (mIU/dl) | 304.8  (141.8-406.5) | 287.2  (129.0-582.6) | 325.9  (201.8-953.4) | 229.8  (17.9-2146) |
| mGDF15 (pg/ml) | 612.4  (272.3-1373.6) | 1415.1  (690.3-5020.9) | 1929.0  (860.7-5840.1) | 3737.1  (1157.3-20910.5) |
| GDPP (ng/ml) | 3.0  (1.6-11.2) | 4.8  (1.9-7.8) | 8.3  (2.7-21.0) | 18.0  (4.4-247.9) |
| Cohort 2 | | | | |
|  | Healthy donors  n=15 | Patients with local PCa  n=30 | Patients with mCRPC (BM-)  n=7 | Patients with mCRPC (BM+)  n=40 |
| Age (years) | 62  (43-74) | 67.5  (52-77) | 72  (65-83) | 74  (51-87) |
| Gleason Score  <8  ≥8 | - | 27  3 | 3  4 | 11  29 |
| pT stage pT2  pT3 | - | 25  5 | - | - |
| PSA (ng/ml) | 1.5  (0.4-8.5) | 8.0  (3.2-38.3) | 5.8  (0-52.9) | 14.6  (0-2552) |
| ALP (U/l) | 186  (45-279) | 204.5  (123-442) | 210  (65-237) | 188.5  (37-1815) |
| LDH (U/l) | 161  (118-340) | 175.5  (135-252) | 176  (151-280) | 193  (132-664) |
| OC (ng/ml) | 15.5  (4.5-22.0) | 14.7  (4.8-33.6) | 18.1  (6.1-38.3) | 9.9  (1.4-80.7) |
| BAP (μg/l) | 17.7  (13.9-24.4) | 22.1  (12.8-49.4) | 18.8  (14.9-24.0) | 20.5  (0.8-364.9) |
| PⅠNP (ng/ml) | 11.9  (6.6-24.6) | 34.7  (18.4-72.2) | 123.9  (21.5-192.2) | 35.7  (3.9-189.1) |
| TRACP 5b (mIU/dl) | 284.5  (141.8-532.9) | 250.3  (117.1-582.5) | 406.5  (159.9-654.8) | 243.0  (50.6-2795.4) |
| mGDF15 (pg/ml) | 684.0  (402.3-1802.9) | 1246.7  (632.9-2246.3) | 1693.0  (1024.7-7266.1) | 3622.9  (760.0-21351.5) |
| GDPP (ng/ml) | 2.6  (2.1-6.9) | 4.4  (2.5-7.9) | 10.5  (4.4-15.8) | 14.0  (4.4-168.5) |

**Table S4.** Comparison of the utility of blood biomarkers in CRPC patients with BM.

| Biomarker | Recall rate | F1 score | Precision | Specificity | Sensitivity |
| --- | --- | --- | --- | --- | --- |
| Cohort 1 | | | | | |
| GDPP | 82.5% | 0.836 | 84.6% | 88.7% | 82.5% |
| mGDF15 | 72.5% | 0.795 | 87.9% | 92.4% | 72.5% |
| PSA | 75.5% | 0.803 | 85.7% | 92.5% | 75.5% |
| BAP | 67.5% | 0.759 | 86.7% | 96.2% | 67.5% |
| PINP | 35.0% | 0.467 | 70.0% | 88.7% | 35.0% |
| LDH | 50.0% | 0.625 | 83.3% | 92.5% | 50.0% |
| Cohort 2 | | | | | |
| GDPP | 82.5% | 0.836 | 84.6% | 88.5% | 82.5% |
| mGDF15 | 80.0% | 0.762 | 72.7% | 76.9% | 80.0% |
| PSA | 62.5% | 0.769 | 100.0% | 100.0% | 62.5% |
| BAP | 35.0% | 0.444 | 60.9% | 82.7% | 35.0% |
| PINP | 40.0% | 0.470 | 57.1% | 71.2% | 40.0% |
| LDH | 72.5% | 0.690 | 65.9% | 71.2% | 72.5% |

**Table S5.** Background of CRPC patients with BM whose blood samples were collected over time.

|  | mCRPC (BM+)  n=22 |
| --- | --- |
| Age (years) | 73 (61-84) |
| Gleason Score <8  ≥8 | 2  20 |
| Duration between blood samples (months) | 26.5 (6-66) |
| Number of systemic therapies | 2 (1-5) |
| Type of systemic therapy | Bicalutamide 7  Flutamide 7  Enzalutamide 11  Abiraterone 1  Docetaxel 9  Cabazitaxel 4  Radium-223 5 |

**Table S6.** Univariate and multivariate logistic regression analysis of CSS in CRPC patients with BM (n=80)

|  | Univariate analysis | | | Multivariate analysis | | |
| --- | --- | --- | --- | --- | --- | --- |
|  | HR | 95% CI | *P value* | HR | 95% CI | *P value* |
| Age  (<75 vs. ≥75 y) | 0.80 | 0.37-1.72 | 0.56 | - | - | - |
| Gleason Score  (<8 vs. ≥8) | 0.59 | 0.24-1.42 | 0.24 | - | - | - |
| PSA (ng/ml)  (<17.7 vs. ≥17.7) | 10.4 | 3.67-29.3 | <0.01 | 6.61 | 2.01-21.7 | <0.01 |
| ALP (U/l)  (<186.5 vs. ≥186.5) | 1.21 | 0.55-2.64 | 0.64 | - | - | - |
| LDH (U/l)  (<210.5 vs. ≥210.5) | 1.53 | 0.70-3.33 | 0.28 | - | - | - |
| OC (ng/ml)  (<7.8 vs. ≥7.8) | 0.64 | 0.29-1.39 | 0.26 | - | - | - |
| BAP (μg/l)  (<22.5 vs. ≥22.5) | 2.53 | 1.12-5.67 | 0.025 | 1.56 | 0.63-3.85 | 0.34 |
| PⅠNP (ng/ml)  (<37.0 vs. ≥37.0) | 1.10 | 0.51-2.37 | 0.81 | - | - | - |
| TRACP 5b (mIU/dl)  (<237 vs. ≥237) | 1.49 | 0.68-3.25 | 0.32 | - | - | - |
| GDPP (ng/ml)  (<15.3 vs. ≥15.3) | 11.0 | 3.96-30.3 | <0.01 | 7.03 | 2.30-21.5 | <0.01 |

CI; confidence interval, HR; hazard ratio

**Table S7.** Patient characteristics and blood biomarkers of HSPC patients. All continuous data are presented with median value and range.

|  | Healthy donors  n=30 | Local PCa  n=60 | mHSPC (BM+)  n=30 |
| --- | --- | --- | --- |
| Age (years) | 63  (37-74) | 69  (52-79) | 72  (51-84) |
| Gleason Score  ＜8  ≧8 | - | 53  7 | 2  28 |
| pT stage  pT2  pT3 | - | 48  12 | - |
| Metastasis site | - | - | Bone: 30  Lymph node: 8  Lung: 3 |
| PSA (ng/ml) | 0.96  (0-8.5) | 6.5  (2.6-45.5) | 376.1  (0-6699) |
| ALP (U/l) | 183  (45-292) | 196  (107-442) | 188.5  (50-1281) |
| LDH (U/l) | 172  (118-340) | 175  (120-312) | 191  (126-422) |
| OC (ng/ml) | 15.2  (4.5-22.0) | 14.7  (3.6-35.8) | 16.6  (3.6-81.0) |
| BAP (μg/l) | 18.4  (13.9-30.7) | 22.2  (10.5-49.4) | 42.4  (12.0-341.1) |
| PⅠNP (ng/ml) | 11.9  (6.5-24.6) | 36.4  (14.8-72.2) | 64.4  (10.2-247.5) |
| TRACP 5b (mIU/dl) | 286.2  (141.8-532.9) | 276.0  (117.1-582.6) | 542.8  (116.3-2034) |
| mGDF15 (pg/ml) | 626.7  (272.3-1802.9) | 1331.3  (632.9-5020.9) | 5809.9  (995.5-21576.0) |
| GDPP (ng/ml) | 2.8  (1.6-11.2) | 4.5  (1.9-7.9) | 13.0  (4.7-225.3) |

**Table S8.** Comparison of the utility of blood biomarkers in HSPC patients with BM.

| Biomarker | Recall rate | F1 score | Precision | Specificity | Sensitivity |
| --- | --- | --- | --- | --- | --- |
| GDPP | 100.0% | 0.667 | 50.0% | 66.7% | 100.0% |
| mGDF15 | 66.7% | 0.755 | 87.0% | 96.7% | 66.7% |
| PSA | 83.3% | 0.877 | 92.6% | 97.8% | 83.3% |
| OC | 26.7% | 0.381 | 66.7% | 95.6% | 26.7% |
| BAP | 53.3% | 0.653 | 84.2% | 96.7% | 53.3% |
| PINP | 63.3% | 0.678 | 73.1% | 92.2% | 63.3% |
| TRACP 5b | 76.7% | 0.613 | 51.1% | 75.6% | 76.7% |
| ALP | 40.0% | 0.511 | 70.6% | 94.4% | 40.0% |
| LDH | 33.3% | 0.444 | 66.7% | 94.4% | 33.3% |

**Table S9.** Univariate and multivariate analyses of BM diagnostic biomarkers in PCa patients using logistic regression analysis (BM-: n=75, BM+: n=110).

|  | Univariate analysis | | | Multivariate analysis | | |
| --- | --- | --- | --- | --- | --- | --- |
|  | OR | 95% CI | *P value* | OR | 95% CI | *P value* |
| PSA (ng/ml)  (<11.7 vs. ≥11.7) | 4.68 | 2.47-8.86 | <0.01 | 1.61 | 0.72-3.61 | 0.25 |
| ALP (U/L)  (<190 vs. ≥190) | 0.86 | 0.48-1.54 | 0.61 | - | - | - |
| LDH (U/L)  (<187 vs. ≥187) | 2.57 | 1.40-4.71 | <0.01 | 1.50 | 0.71-3.20 | 0.29 |
| OC (ng/ml)  (<12.9 vs. ≥12.9) | 0.38 | 0.20-0.69 | <0.01 | - | - | - |
| BAP (μg/l)  (<23.0 vs. ≥23.0) | 1.94 | 1.07-3.52 | 0.030 | 1.45 | 0.68-3.06 | 0.33 |
| PINP (ng/ml)  (<41.8 vs. ≥41.8) | 1.12 | 0.62-2.02 | 0.70 | - | - | - |
| TRACP 5b (mIU/dl)  (<298.8 vs. ≥298.8) | 1.23 | 0.68-2.21 | 0.49 | - | - | - |
| GDPP (ng/ml)  (<7.7 vs. ≥7.7) | 19.0 | 8.62-42.0 | <0.01 | 14.0 | 5.98-32.8 | <0.01 |

OR; odds ratio, CI; confidence interval

**Table S10.** Background of localized prostate cancer patients who underwent radical prostatectomy. All continuous data are presented with median value and range.

|  | Local PCa  n=179 |
| --- | --- |
| Age (years) | 68 (51-75) |
| PSA (ng/ml) | 7.0 (3.4-42.7) |
| Gleason Score <8  ≥8 | 153  26 |
| pT stage pT2  pT3 | 137  42 |
| ALP (U/I) | 200 (84-442) |
| LDH (U/I) | 176 (120-384) |
| GDPP (ng/ml) | 1.5 (0.2-6.8) |
