# Supplementary figures and images for: GDF15 propeptide promotes bone metastasis of castration-resistant prostate cancer by augmenting the bone microenvironment
Source: Biomark Res. 2024 Nov 25;12:147. doi: 10.1186/s40364-024-00695-6 (PMC11590406; doi:10.1186/s40364-024-00695-6)

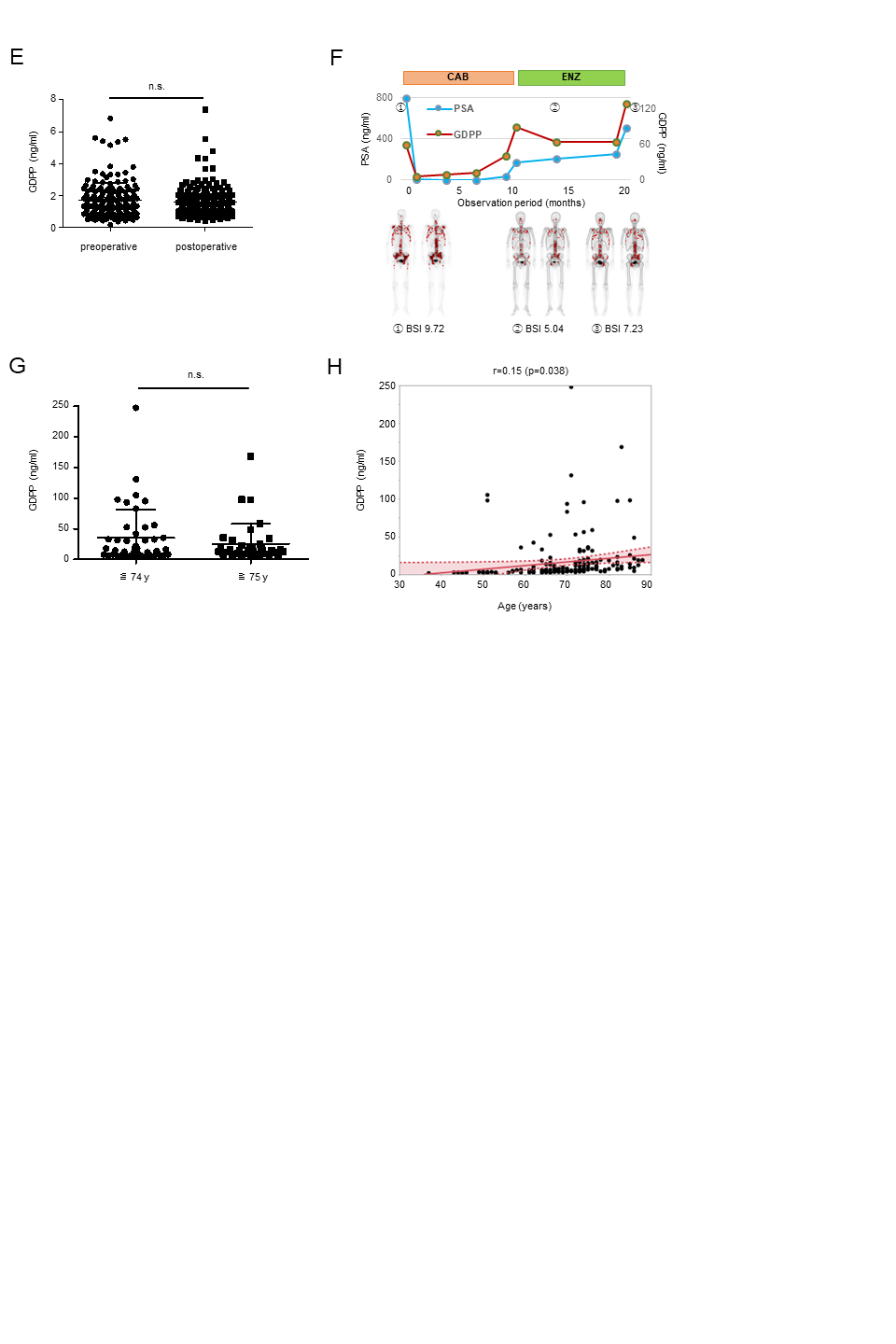

Supplement: Supplementary file 2 — Supplementary Material 2 [file 40364_2024_695_MOESM2_ESM.tif]

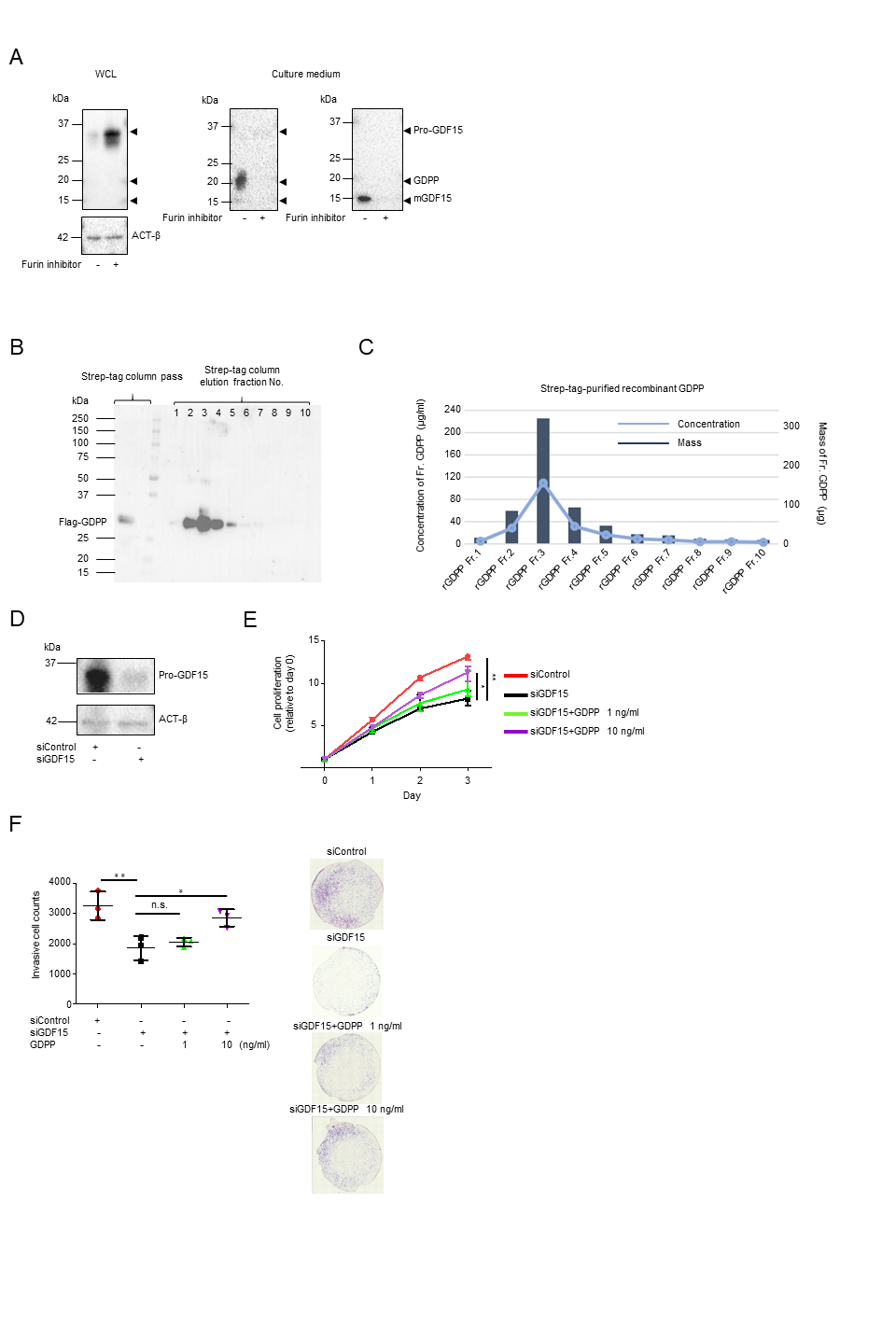

Supplement: Supplementary file 3 — Supplementary Material 3 [file 40364_2024_695_MOESM3_ESM.tif]

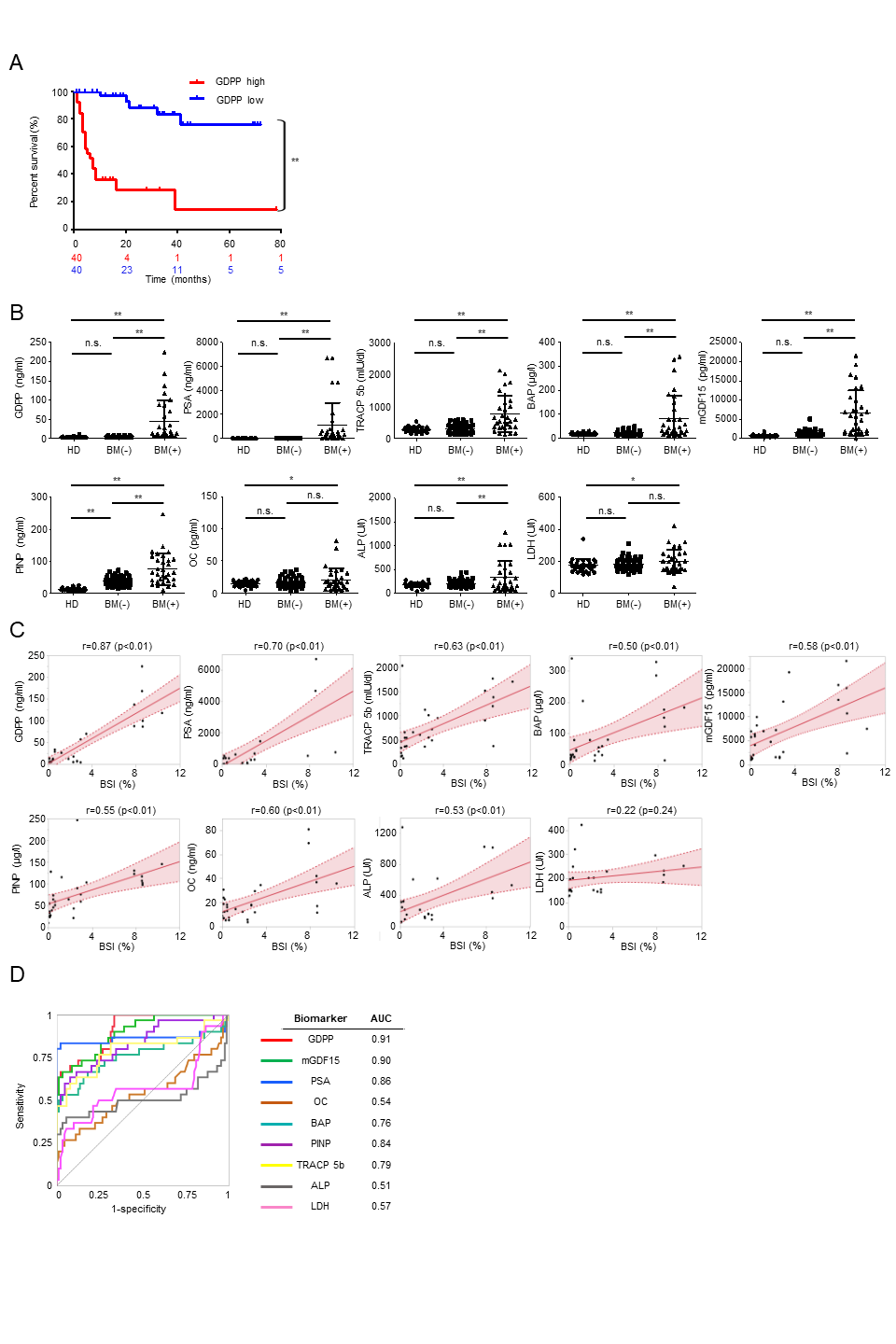

Supplement: Supplementary file 4 — Supplementary Material 4 [file 40364_2024_695_MOESM4_ESM.tif]

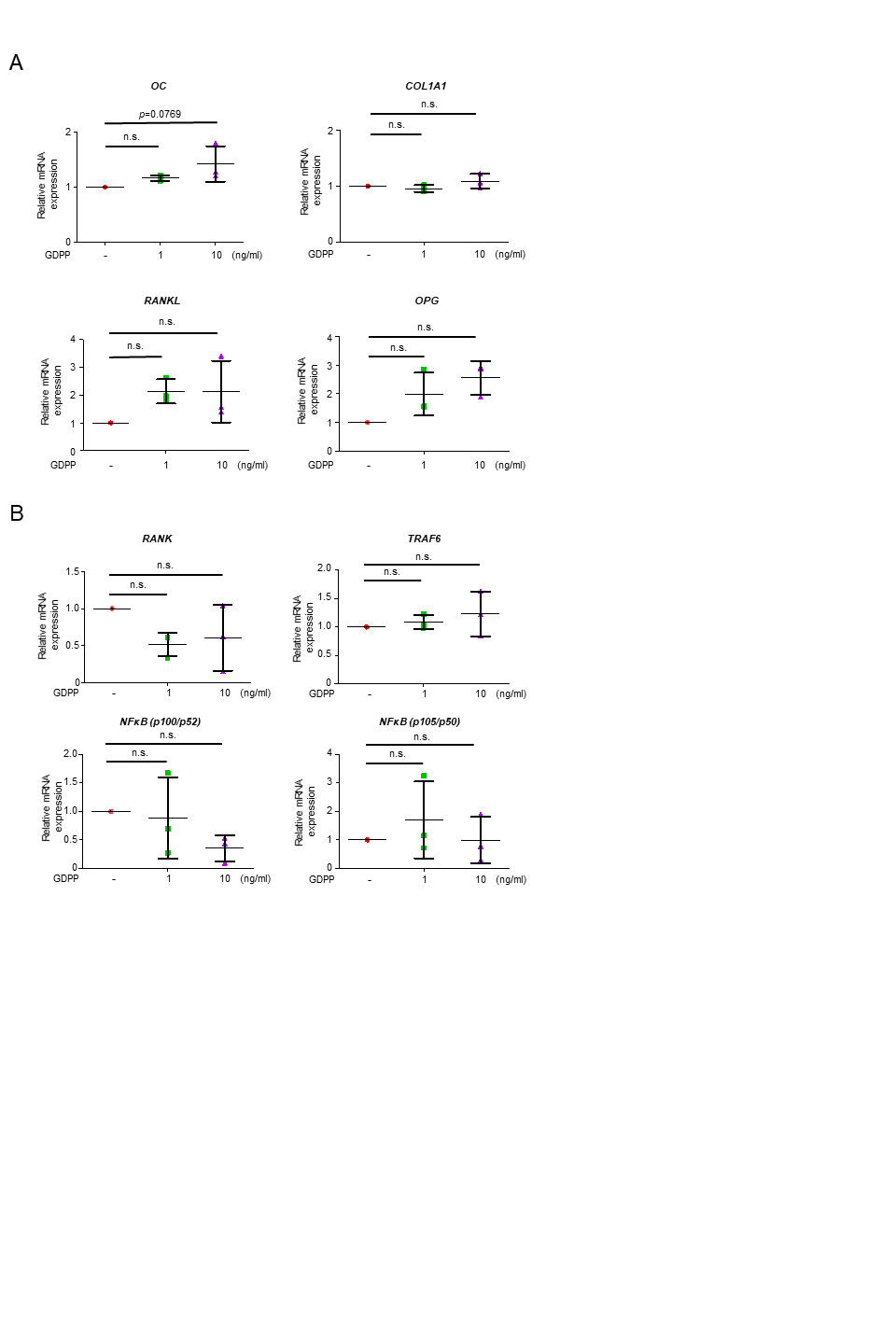

Supplement: Supplementary file 5 — Supplementary Material 5 [file 40364_2024_695_MOESM5_ESM.tif]

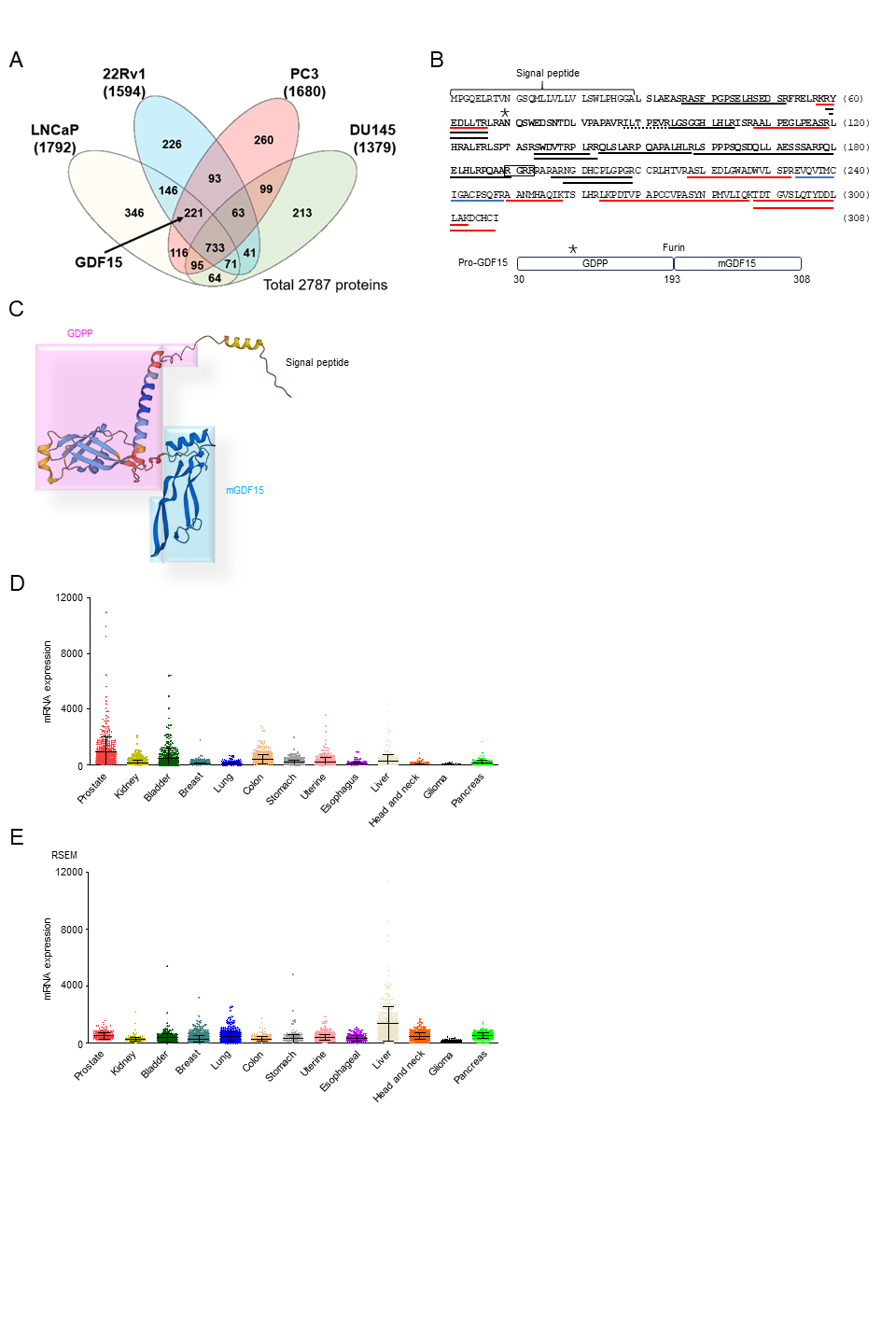

Supplement: Supplementary file 6 — Supplementary Material 6 [file 40364_2024_695_MOESM6_ESM.tif]

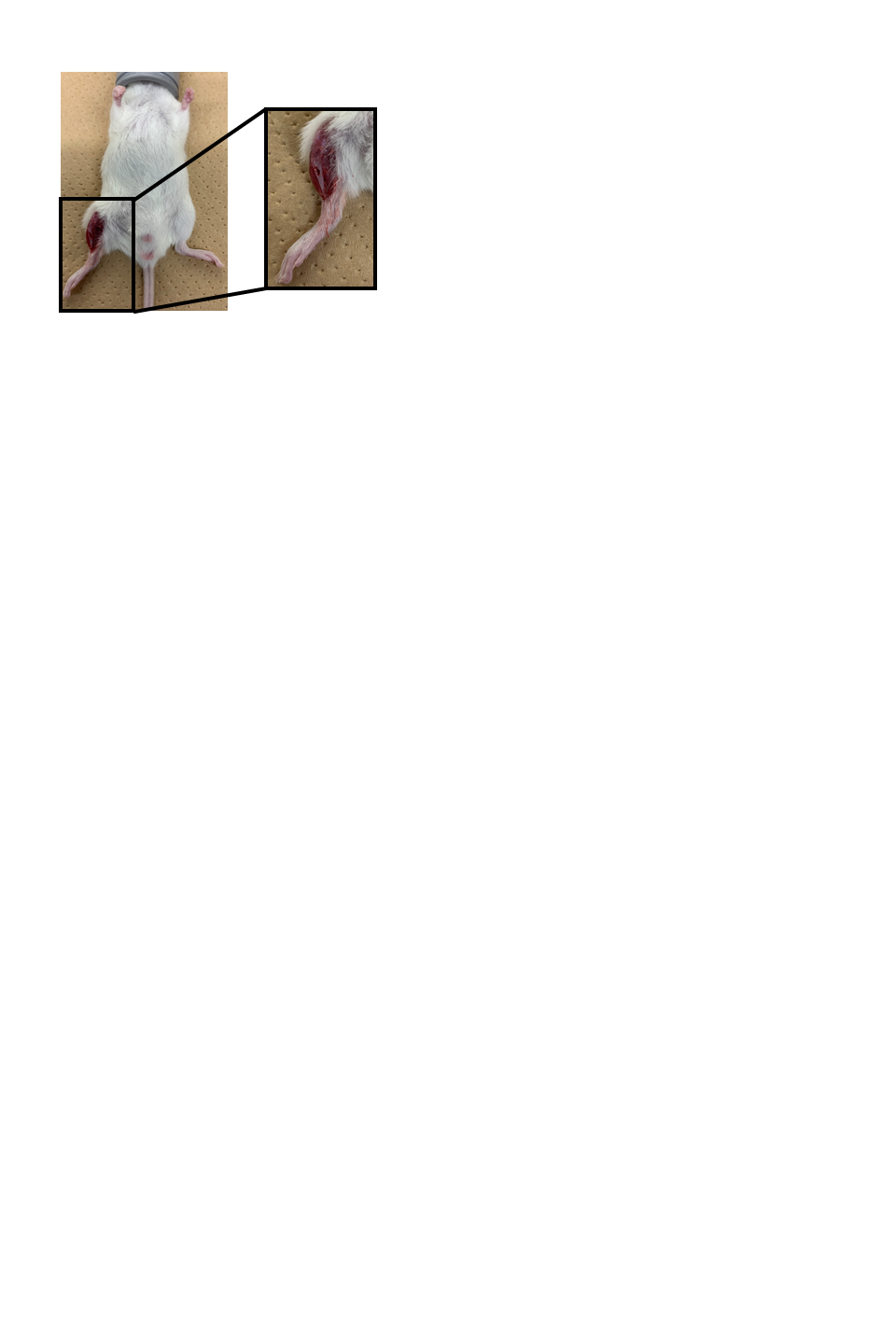

Supplement: Supplementary file 7 — Supplementary Material 7 [file 40364_2024_695_MOESM7_ESM.tif]

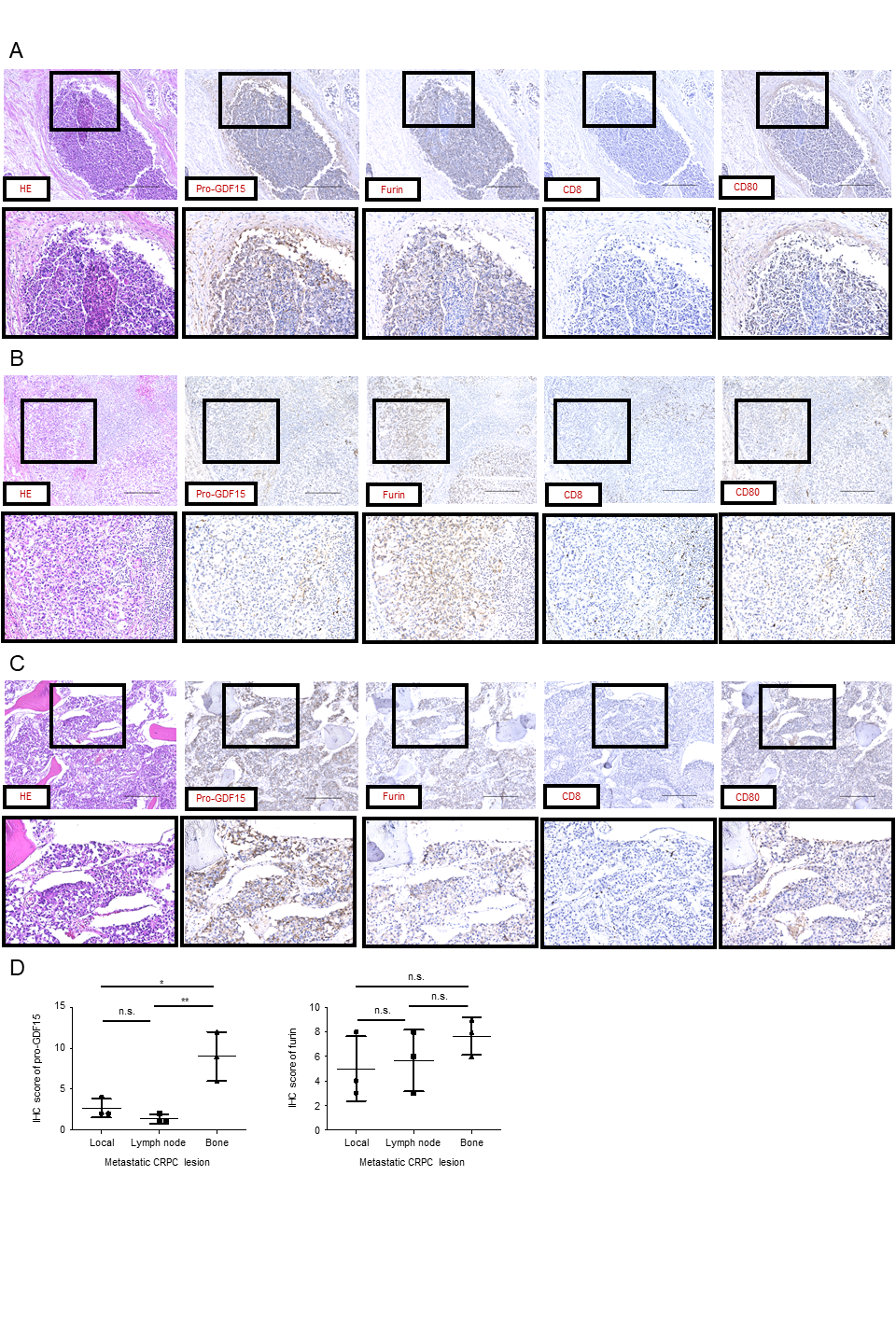

Supplement: Supplementary file 8 — Supplementary Material 8 [file 40364_2024_695_MOESM8_ESM.tif]
